# Solving the puzzling competition of the thermal C^2^–C^6^ vs Myers–Saito cyclization of enyne-carbodiimides

**DOI:** 10.3762/bjoc.12.6

**Published:** 2016-01-11

**Authors:** Anup Rana, Mehmet Emin Cinar, Debabrata Samanta, Michael Schmittel

**Affiliations:** 1 Department of Chemistry and Biology, Universität Siegen, Adolf-Reichwein-Str. 2, D-57068 Siegen, Germany

**Keywords:** DFT computation, diradical, enyne-carbodiimides, hydrogen transfer, thermal cyclization

## Abstract

The mechanism of the thermal cyclization of enyne-carbodiimides **7a–c** has been studied computationally by applying the DFT method. The results indicate that enyne-carbodiimides preferentially follow the C^2^–C^6^ (Schmittel) cyclization pathway in a concerted fashion although the Myers–Saito diradical formation is kinetically preferred. The experimentally verified preference of the C^2^–C^6^ over the Myers–Saito pathway is guided by the inability of the Myers–Saito diradical to kinetically compete in the rate-determining trapping reactions, either inter- or intramolecular, with the concerted C^2^–C^6^ cyclization. As demonstrated with enyne-carbodiimide **11**, the Myers–Saito channel can be made the preferred pathway if the trapping reaction by hydrogen transfer is no more rate determining.

## Introduction

**Scheme 1:**
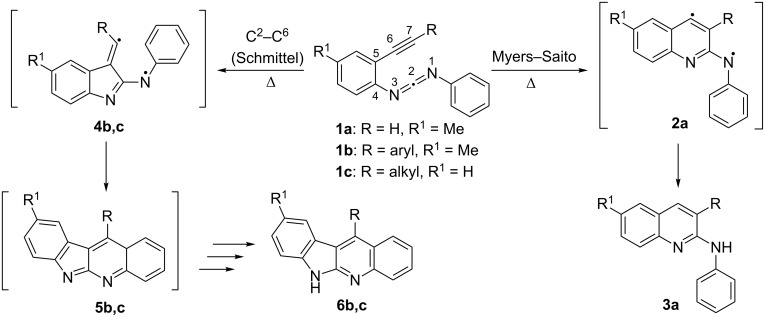
Thermal cyclization modes of enyne-carbodiimides **1a–c**.

The thermal cyclizations of enediynes [1–6], enediallenes [7–10], bisallenes [11], enyne-allenes [12–17], and enyne-carbodiimides [18–25] have received great interest over the last two decades due to their value for organic synthesis and their interesting mechanistic facets [26]. Like for enyne-allenes, the cyclization mode of enyne-carbodiimides **1** (Scheme 1) can be switched between C^2^–C^6^ and Myers–Saito reaction channels depending on the substitution at the alkyne terminus, as disclosed by Schmittel [18] and Wang [19]. In 1998, Schmittel et al. [18] reported that the thermolysis of enyne-carbodiimide **1a** in toluene/1,4-cyclohexadiene yielded the Myers–Saito product **3a**, whereas the thermolysis of enyne-carbodiimide **1b** produced the C^2^–C^6^ product **6b**. In 1999 Wang et al. [19] stated that the thermal cyclization of enyne-carbodiimides **1c** furnished C^2^–C^6^ (Schmittel) products **6c** in *p*-xylene under reflux conditions. Based on the experimental findings it was postulated that the cyclization mode was shifted to the C^2^–C^6^ channel if more sterically demanding substituents than hydrogen were attached to the alkyne terminus, because the resulting strong repulsion of *ortho*-substituents in diradical **2b,c** should disfavor the Myers–Saito pathway. At that time, diradical intermediates were invoked for both pathways based on analogy and computational results [18]. To obtain a deeper understanding of this switch in selectivity, we decided to amalgamate computations and new mechanistic experiments to resolve the puzzle.

**Scheme 2:**
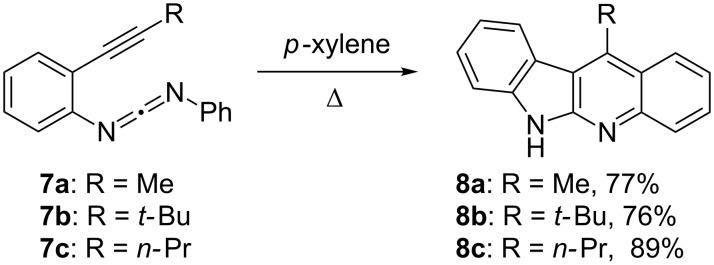
Thermolysis of enyne-carbodiimides **7a–c** furnishing **8a–c** [19].

For the computational part of this study, we chose first to interrogate enyne-carbodiimides **7a–c** (Scheme 2), the synthesis and thermolysis of which have been reported by Wang and coworkers [19]. The methyl (**7a**) and *tert*-butyl groups (**7b**) at the alkyne terminus are ideal to vary the steric influence and the *n*-propyl group in **7c** offers a realistic option for an intramolecular γ-hydrogen abstraction via a six-membered transition state if the diradical is thermally generated. From the computations (vide infra) we conclude that the thermal cyclization of enyne-carbodiimides **7** follows a concerted mechanism along the C^2^–C^6^ (Schmittel) channel and the decision between the two reaction channels for **7** is not necessarily guided by the free activation energy of the two initial steps, but by the inability of the Myers–Saito diradical to compete in follow-up reactions, either inter- or intramolecular, with the C^2^–C^6^ (Schmittel) channel.

## Results and Discussion

The proper description of organic reactions involving diradical intermediates and transition state structures remains a difficult task in quantum chemistry [27–35]. Singlet diradicals have two electronic configurations that can only be treated accurately by costly multi-reference techniques. An alternative is to apply the unrestricted broken symmetry (U–BS) density functional theory (DFT) approach, which is often in use for larger systems due to its good results [36–49]. Hence, we applied the U–BS DFT approach for the diradical species and the transition states (TSs) connecting them while the restricted method was used for closed shell species. The BLYP functional was chosen for computation because the benchmark studies done by Schreiner showed good applicability of BLYP for the enyne-allene system [3]. Because nitrogen atoms are present in the cumulene core, the enyne-carbodiimide system is expected to be polar and that is why the 6-31+G* basis set was used. All computations were performed in the Gaussian 09 [50] program package. As the reactions take place in a nonpolar solvent (*p*-xylene), calculations were carried out in vacuum. The nature of all the stationary points was confirmed by frequency analysis. The intrinsic reaction coordinate (IRC) was followed in mass-scaled Cartesian coordinates to validate the TSs. The stability of the wave functions was tested with the stable=opt keyword.

**Figure 1:**
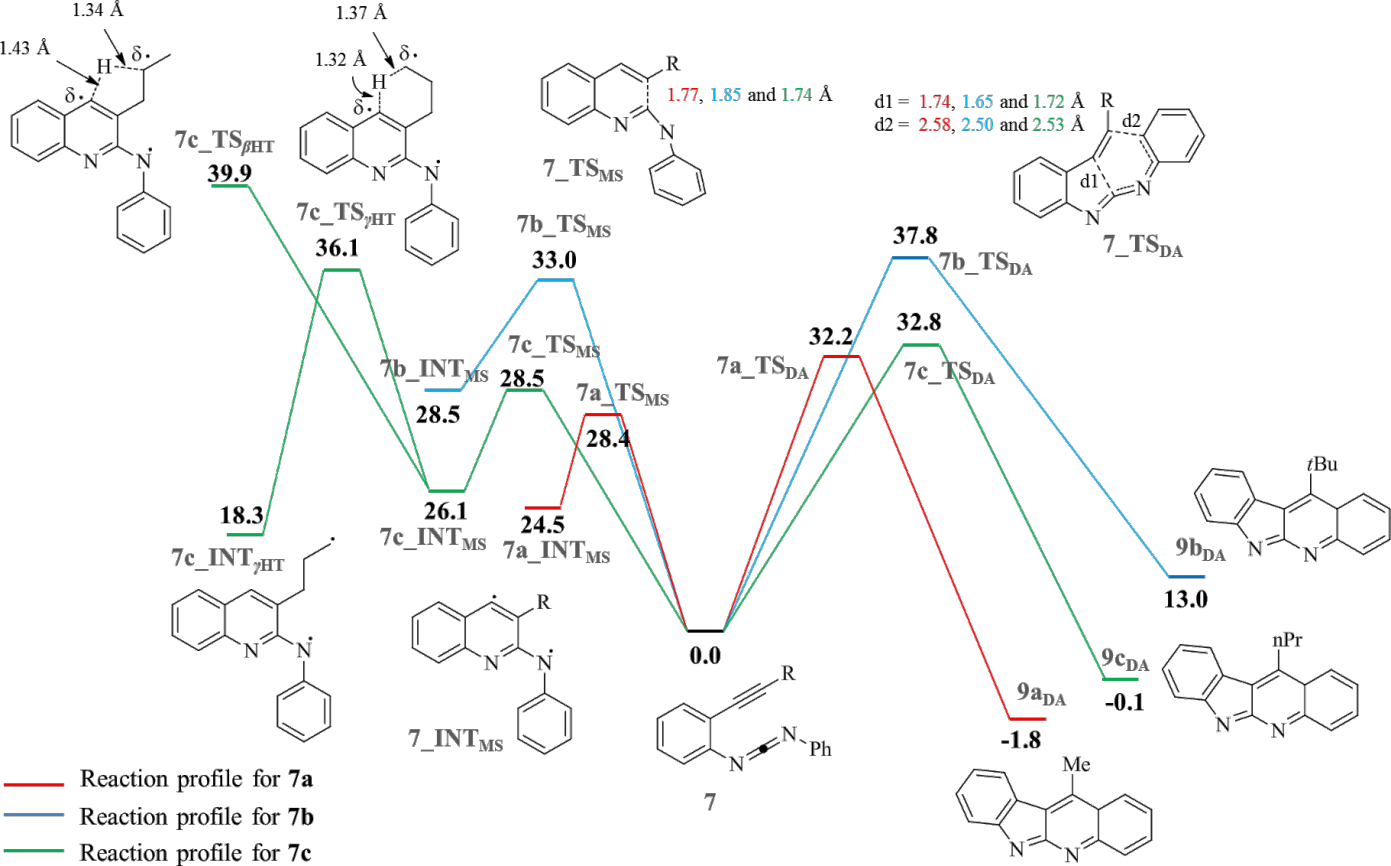
Reaction profile of **7a–c** at BLYP/6-31+G* level. All bold numbers represent the respective free energies in kcal mol**^–^**^1^ at 138 °C. Subscripts “_DA_”, “_MS_” and “_HT_” stand for “Diels–Alder”, “Myers–Saito” and “Hydrogen Transfer”, respectively.

In the computations, we were unable to locate any diradical intermediate along the C^2^–C^6^ (Schmittel) channel. Rather the results indicate that all enyne-carbodiimides **7a–c** follow a concerted intramolecular Diels–Alder (DA) pathway toward the DA products **9_DA_** (Figure 1), which subsequently rearrange to the isolated final products **8a–c** thus avoiding a C^2^–C^6^ diradical mechanism. As expected due to the strong steric shielding about the *tert*-butyl group, enyne-carbodiimide **7b** exhibits the highest free energy barrier, i.e., 37.8 kcal mol^−1^ at 138 °C (= the reaction temperature). The free energies of activation for enyne**-**carbodiimides **7a** and **7c** are significantly lower at 32.2 and 32.8 kcal mol^−1^, respectively. Disturbingly, though, the computations predict in all cases a strong kinetic preference for the Myers–Saito (Figure 1) over the concerted C^2^–C^6^ (DA) pathway. The C^2^–C^6^ Diels–Alder TS **7a_TS_DA_** is 3.8 kcal mol^−1^ higher in energy than the corresponding Myers–Saito TS **7a_TS_MS_**, while the difference is 4.8 and 4.3 kcal mol^−1^ for **7b** and **7c**, respectively. Thus, at this point, the computational results completely contradict the experimental findings. One plausible explanation for the exclusive formation of **8** is that in absence of any efficient hydrogen donor, the kinetically preferred Myers–Saito diradical intermediate **7_INT_MS_** has to be in a fully reversible equilibrium with the starting material **7**, so that eventually product **9** will form. Indeed, at the stage of the Myers–Saito diradical intermediate **7c_INT_MS_** there are two intramolecular radical quenching processes possible and both exhibit a higher lying barrier than that of the C^2^–C^6^ cyclization **7c_TS_DA_** (Δ*G*^‡^ = 32.8 kcal mol^−1^ – referenced to **7c**). The first one invokes a γ–H abstraction at the propyl chain of **7c_INT_MS_** via the six-membered cyclic TS **7c_TS_γHT_** (Δ*G*^‡^ = 36.1 kcal mol^−1^) and the second possibility an intramolecular β–H abstraction via **7c_TS_βHT_** (Δ*G*^‡^ = 39.9 kcal mol^−1^). As a consequence, enyne-carbodiimide **7c** preferentially cyclizes via the C^2^–C^6^ pathway to **9c_DA_**, because the Myers–Saito route with its rate-limiting γ- or β-hydrogen abstraction steps is disfavored on the free energy surface (ΔΔ*G*^‡^ = 3.3 and 7.1 kcal mol^−1^).

**Scheme 3:**
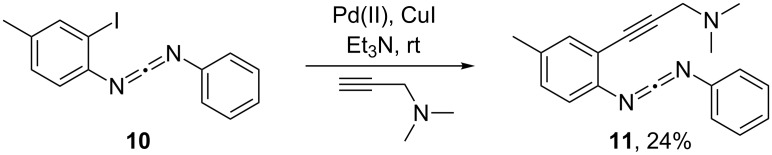
Synthesis of enyne-carbodiimide **11**.

After the insightful computational analysis of the reaction mechanism of **7a–c**, we chose to use computations in search for an enyne-carbodiimide with a preferred Myers–Saito pathway that would not carry hydrogen at the alkyne terminus. For any suitable candidate the intermediate Myers–Saito diradical should undergo intramolecular hydrogen abstraction at a lower barrier than that of the concerted C^2^–C^6^ cyclization. In order to realize a facile intramolecular H-abstraction in the Myers–Saito diradical via a 6-membered transition state [26,45], we decided to implement the *N*,*N*-dimethylaminomethyl group at the alkyne terminus. Two factors should be favorable for effective hydrogen transfer: (i) the nitrogen atom [51] stabilizes the incipient radical center and (ii) six equivalent hydrogens are available for transfer. Radical stabilization by heteroatoms (with lone pairs) has recently gained interest as a reaction design tool [52–53]. Thus, enyne-carbodiimide **11** with its CH_2_NMe_2_ group was selected as a target and prepared (vide infra) according to Scheme 3.

**Scheme 4:**
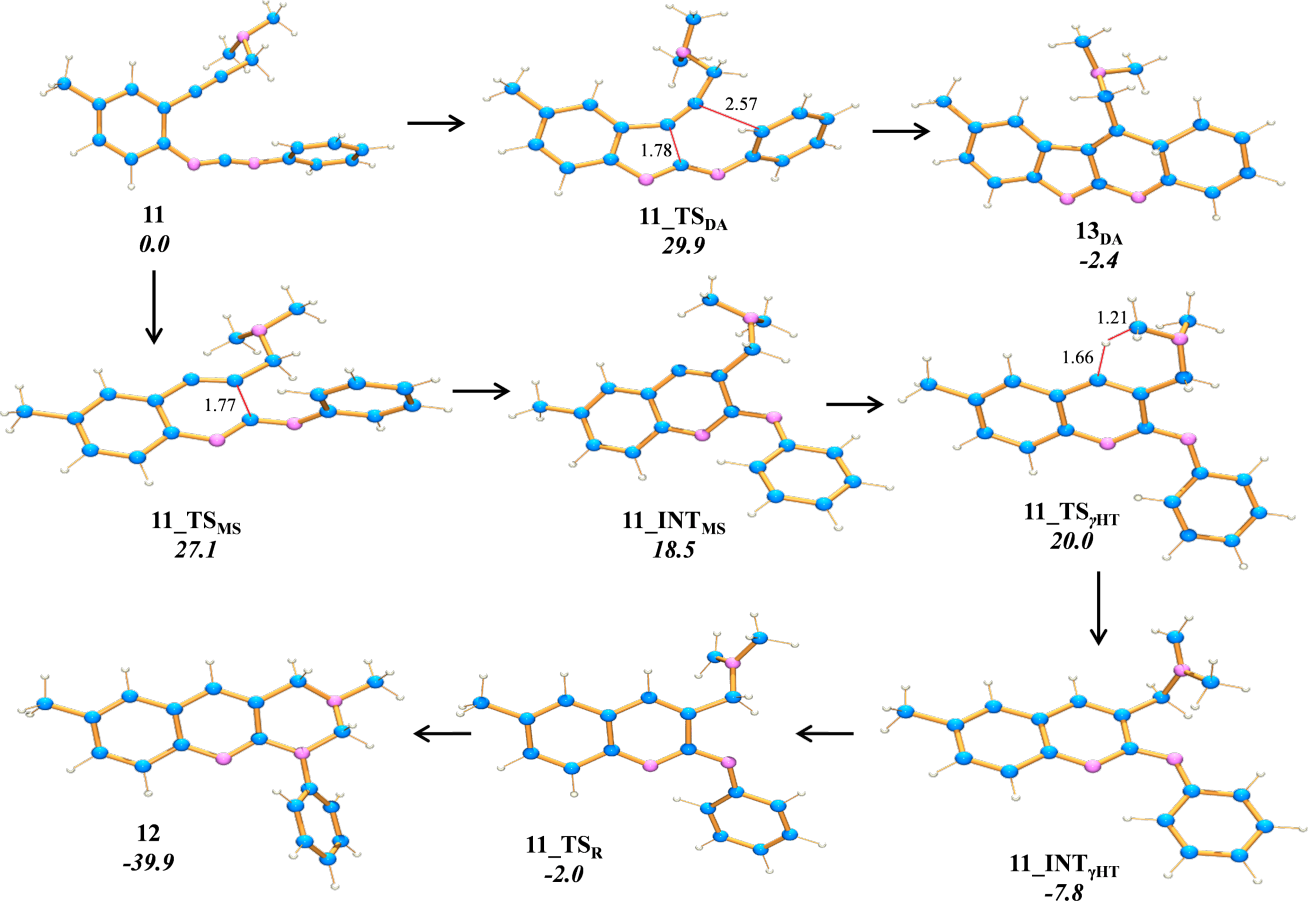
Energetics of the thermal cyclization of enyne-carbodiimide **11** at BLYP/6-31+G* level. Bold numbers in italics are representing respective free energies in kcal mol**^–1^** at 25 °C and other numbers are representing bond distances in angstrom (Å). Subscripts “_DA_”, “_MS_”, “_HT_” and “_R_” stand for “Diels–Alder”, “Myers–Saito”, “Hydrogen Transfer” and “Rotation”, respectively.

Computations at the BLYP/6-31+G* level of theory on the thermal cyclization of **11** (Scheme 4) provided a barrier **11_TS_DA_** of 29.9 kcal mol^−1^ for the concerted C^2^-C^6^ DA cyclization toward **13_DA_**. Notably, both the Myers–Saito diradical cyclization step via **11_INT_MS_** (27.1 kcal mol^−1^) and the follow-up intramolecular hydrogen abstraction **11_INT_MS_** → **11_INT_γHT_** are favored kinetically over formation of **13_DA_**, suggesting that in the thermolysis the Myers–Saito product **12** should prevail. Due to the low free activation energy for the strongly exergonic intramolecular hydrogen abstraction **11_INT_MS_** → **11_INT_γHT_** of only 1.5 kcal mol^−1^, the diradical cyclization step is now rate determining in the Myers–Saito pathway.

**Scheme 5:**
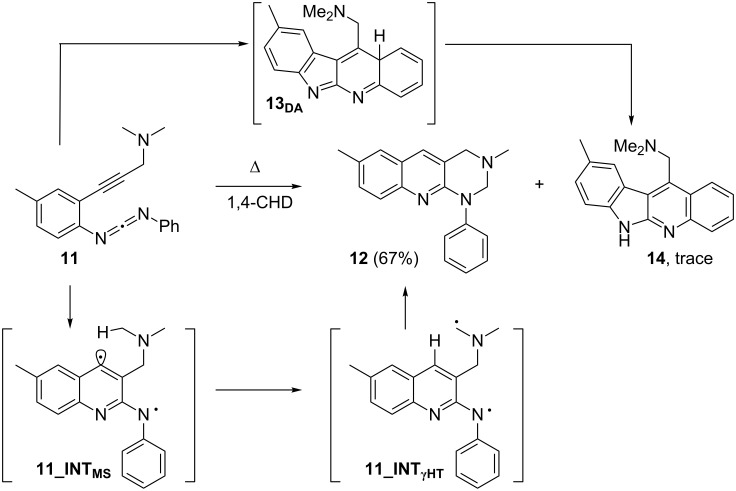
Thermolysis of enyne-carbodiimide **11**.

The synthetic strategy (Scheme 3) was to first prepare carbodiimide **10** [24] according to a literature procedure. In the next step, a Sonogashira coupling between **10** and *N*,*N*-dimethylprop-2-yn-1-amine afforded the desired enyne-carbodiimide **11** in 24% yield. Thermolysis of **11** (Scheme 5) was carried out in presence of 1,4-cyclohexadiene (1,4-CHD) under toluene reflux condition for 12 h. On heating enyne-carbodiimide **11**, the characteristic strong IR absorptions of the carbodiimide unit at 2143 and 2123 cm^−1^ disappeared, which clearly indicated participation of this moiety during the reaction. Conversion of two methyl groups (NMe_2_ in **11**, 2.23 ppm) to one (NMe in **12**, 2.59 ppm) in the ^1^H NMR spectrum suggested the involvement of one methyl group in the cyclization process. In combination with above two findings, we initially identified the Myers–Saito cyclization product **12**, the assignment of which was further confirmed by ^1^H, ^13^C, ^1^H,^1^H-COSY NMR and also by IR spectroscopy and mass spectrometry. For **14**, a broad singlet at 10.02 ppm that is characteristic for its NH proton was observed in the crude NMR mixture. In summary, the Myers–Saito product **12** was produced as major product, 67% yield, whereas only trace amounts of the C^2^–C^6^ cyclization product **14** arising from **13_DA_** were obtained. The experimental finding is thus in full agreement with the computational predictions and supports the hypothesis of the Myers–Saito diradical being a kinetically favored species formed in a reversible side equilibrium with the starting enyne-carbodiimide. The diradical can be trapped in good yield by hydrogen transfer and possibly also other reactions (e.g., addition to unsaturated bonds), if this process has a sufficiently low barrier [23].

## Conclusion

Experimental and computational techniques have been used to shed light on the C^2^–C^6^ vs Myers–Saito competition in the thermal cyclization of enyne-carbodiimides. Although in most cases the C^2^–C^6^ DA cyclization product is furnished exclusively the results suggest that the Myers–Saito diradical intermediate reversibly forms in a kinetically preferred side equilibrium. This diradical intermediate remains a dead-end route as long as there are no powerful inter- or intramolecular hydrogen transfer pathways available. Considering the computational data, however, also other trapping reactions involving the Myers–Saito diradical should be able to compete with the concerted C^2^–C^6^ (DA) pathway, opening new venues for the preparation of heterocycles from carbodiimides [54].

## Experimental

**General methods.** Anhydrous solvents were used to perform reactions under inert atmosphere (argon). Potassium was utilized to dry tetrahydrofuran (THF) and toluene. Triethylamine (Et_3_N) and dichloromethane (DCM) were dried over calcium hydride. Compounds were purified by flash column chromatography (silica gel, 0.035–0.070 mm). To describe NMR signals, the following abbreviations were utilized: s = singlet, d = doublet, t = triplet, dd = doublet of doublets, tt = triplet of triplets, m = multiplet.

**2-(3-(Dimethylamino)prop-1-yn-1-yl)-4-methyl-*N*-((phenylimino)methylene)aniline (11).** To a solution of *N*-phenyl-*N´*-(2-iodo-4-methylphenyl)carbodiimide [55] (**10**, 1.78 g, 5.11 mmol) and *N*,*N*-dimethylprop-2-yn-1-amine (637 mg, 820 μL, 7.66 mmol) in 50 mL of dry NEt_3_ were added PdCl_2_(PPh_3_)_2_ (70.0 mg, 100 μmol) and copper(I) iodide (38.0 mg, 200 μmol) at room temperature under nitrogen atmosphere. The reaction mixture was stirred overnight. Then the solvent was removed under reduced pressure and diethyl ether (40 mL) was added. The suspension was filtered through celite and the filtrate was washed with water and dried over MgSO_4_. After removal of the solvent, column chromatography over silica gel using (*n*-hexane/ethyl acetate, 1:1, *R*_f_ = 0.28) furnished pure product **11** as a dark-green oil. Yield: 354 mg (1.22 mmol, 24%). *T*_onset_ = 119 °C (DSC). ^1^H NMR (400 MHz, CDCl_3_) δ 2.25 (s, 6H), 2.30 (s, 3H), 3.14 (s, 2H), 7.02 (d, ^3^*J* = 8.2 Hz, 1H), 7.07 (dd, ^3^*J* = 8.2 Hz, ^4^*J* = 1.6 Hz, 1H), 7.16 (tt, ^3^*J* = 7.4 Hz, ^4^*J* = 1.2 Hz, 1H), 7.22–7.25 (m, 2H), 7.28 (d, ^4^*J* = 1.6 Hz, 1H), 7.30–7.34 (m, 2H); ^13^C NMR (100 MHz, CDCl_3_) δ 20.7, 44.1, 48.4, 81.7, 92.2, 119.5, 124.2, 124.3, 125.2, 129.4, 130.0, 133.8, 134.1, 135.1, 136.4, 139.3; IR (KBr) 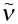: 2976 (w), 2944 (w), 2864 (w), 2825 (w), 2780 (w), 2143 (s, N=C=N), 2123 (s, N=C=N), 1593 (s), 1519 (m), 1488 (s), 1355 (w), 1324 (w), 1284 (w), 1252 (m), 1213 (s), 1034 (m), 908 (s), 822 (m) cm^−1^; EIMS (70 eV) *m*/*z* (%): 289 (29, M^+^), 288 (99, M^+^ − H), 274 (54, M^+^ − CH_3_), 246 (47), 245 (100, M^+^ − N(CH_3_)_2_), 244 (20), 243 (26), 231 (14), 218 (15), 203 (18), 183 (62); ESIMS: calcd. for (C_19_H_20_N_3_)^+^, 290.2; found, 290.2.

**3,7-Dimethyl-1-phenyl-1,2,3,4-tetrahydropyrimido[4,5-*b*]quinoline (12)**. To 10 mL of dry degassed toluene was added 2-(3-(dimethylamino)prop-1-yn-1-yl)-4-methyl-*N*-((phenylimino)methylene)aniline (**11**, 40.0 mg, 138 µmol) and 1,4-CHD (448 mg, 530 μL, 5.60 mmol). The reaction mixture was allowed to reflux for 12 h. After the solvent was removed under reduced pressure, purification was performed by preparative silica gel TLC using *n*-hexane/ethyl acetate (2:3, *R*_f_ = 0.22). Yield 27.0 mg (93.0 µmol, 67%); white solid; mp 124–126 °C; ^1^H NMR (400 MHz, CDCl_3_) δ 2.43 (s, 3H), 2.59 (s, 3H), 4.13 (s, 2H), 4.67 (s, 2H), 7.21 (tt, ^3^*J* = 6.4 Hz, ^4^*J* = 2.1 Hz, 1H), 7.29 (dd, ^3^*J* = 8.6 Hz, ^4^*J* = 2.0 Hz, 1H), 7.33 (br s, 1H), 7.39–7.45 (m, 4H), 7.52 (d, ^3^*J* = 8.6 Hz, 1H), 7.56 (s, 1H); ^13^C NMR (100 MHz, CDCl_3_) δ 21.4, 40.6, 55.8, 73.0, 117.2, 124.3, 125.0, 125.8, 125.8, 126.7, 129.0, 131.0, 132.3, 133.6, 144.3, 145.1, 151.7; IR (KBr) 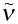: 2945 (m), 2870 (w), 1630 (m), 1594 (m), 1496 (s), 1488 (s), 1455 (m), 1412 (m), 1343 (s, C-N), 1312 (m), 1296 (w), 1225 (w), 1140 (w), 1123 (m), 1068 (m), 1047 (w), 897 (w), 819 (m), 746 (m), 692 (m) cm^−1^; EIMS (70 eV) *m/z* (%): 289 (60, M^+^), 288 (66, M^+^ − H), 245 (100, M^+^ − H − CH_2_NCH_3_), 244 (11), 91 (17); ESI: calcd. for (C_19_H_20_N_3_)^+^, 290.2; found, 290.2.

## Supporting Information

File 1 Characterization and computational data.
