# Supplementary material for: Solving the puzzling competition of the thermal C2–C6 vs Myers–Saito cyclization of enyne-carbodiimides
Source: Beilstein J Org Chem. 2016 Jan 11;12:43–9. doi: 10.3762/bjoc.12.6 (PMC4734315; doi:10.3762/bjoc.12.6)
Supplement: File 1 — Characterization and computational data. [file Beilstein_J_Org_Chem-12-43-s001.pdf]

Supporting Information

for

Solving the puzzling competition of the thermal C^2–C^6 vs Myers–Saito cyclization of enyne-carbodiimides

Anup Rana, Mehmet Emin Cinar, Debabrata Samanta and Michael Schmittel*

Address: Department of Chemistry and Biology, Universität Siegen, Adolf-Reichwein-Str. 2, D-57068 Siegen, Germany

Email: Michael Schmittel - schmittel@chemie.uni-siegen.de

*Corresponding author

Characterization and computational data

Table of contents:

^{1}H and ^{13}C NMR spectra……………………………………………………………….S2–S3

Computational data…………………………………………………………………....S4–S22

^{1}H and ^{13}C NMR spectra:

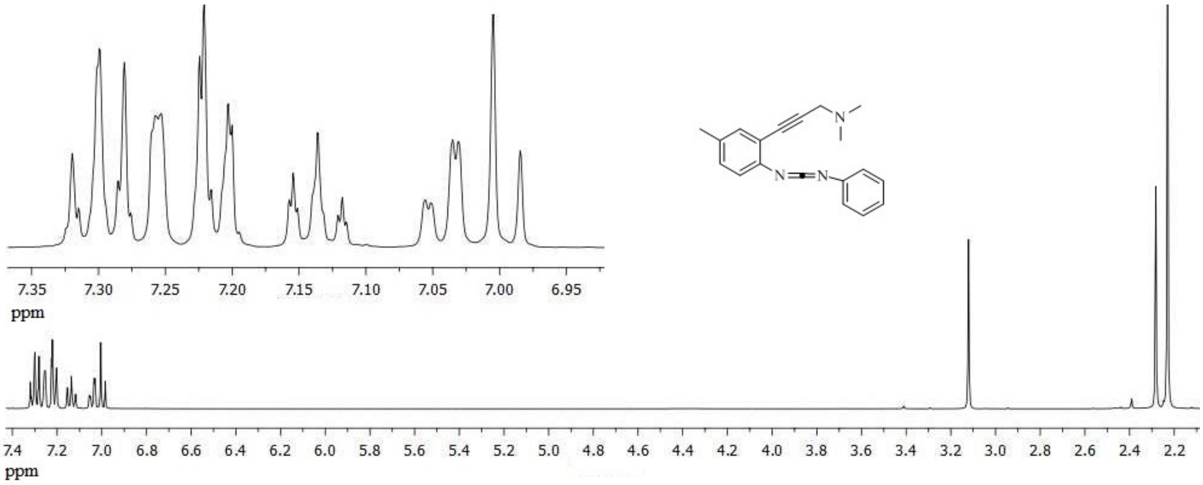

Figure S1. ^{1}H NMR spectrum (400 MHz, $CDCl_3$) of **11**.

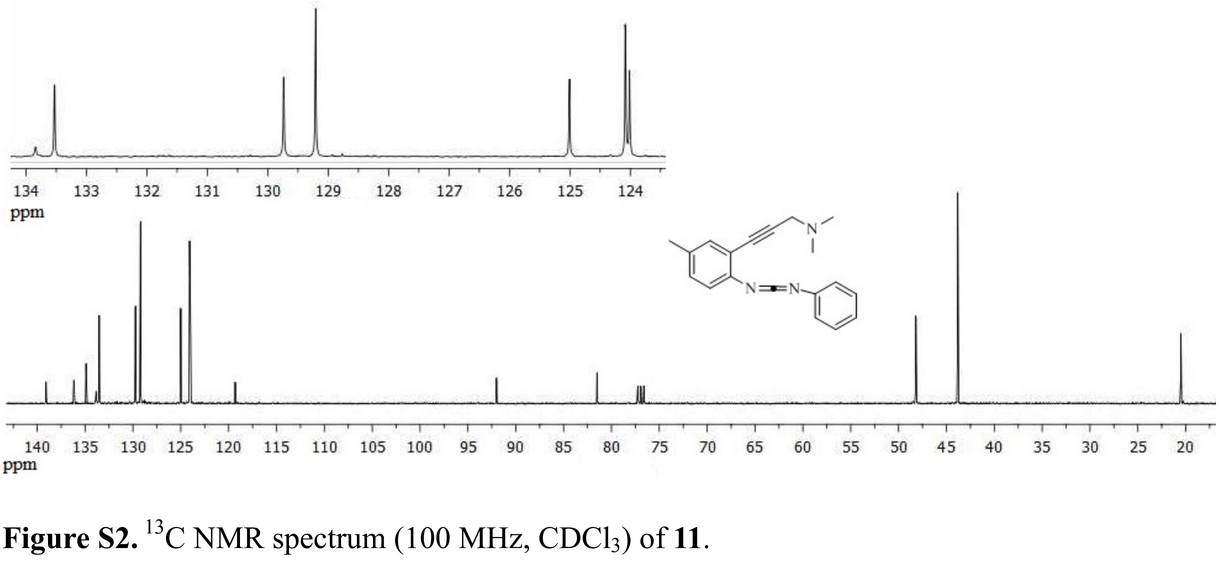

Figure S2. ^{13}C NMR spectrum (100 MHz, $CDCl_3$) of **11**.

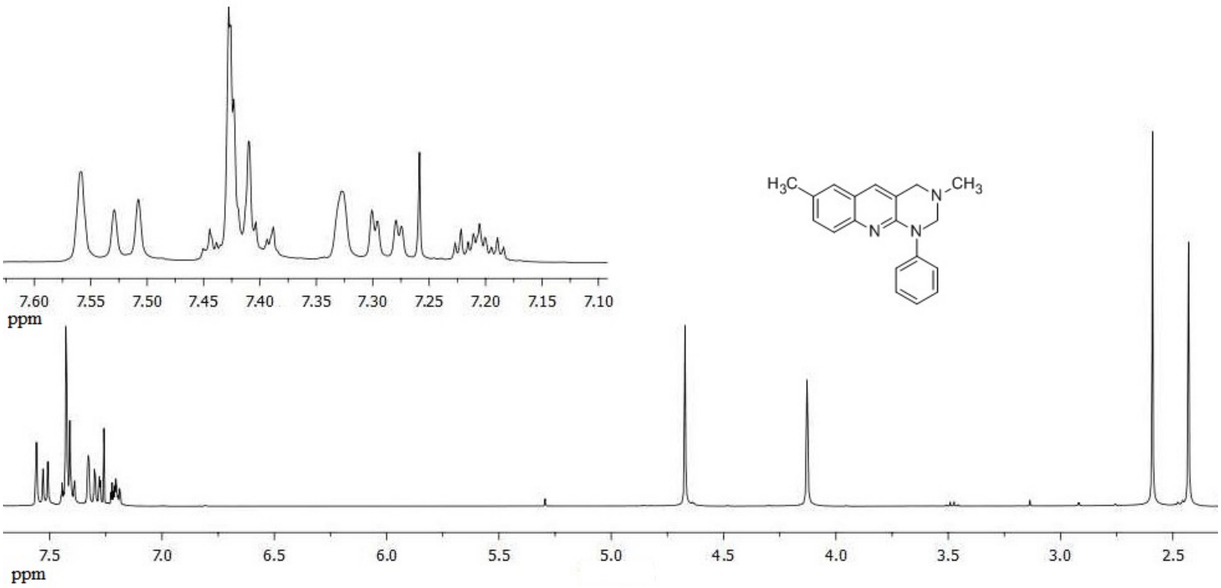

Figure S3. ^{1}H NMR spectrum (400 MHz, $CDCl_3$) of **12**.

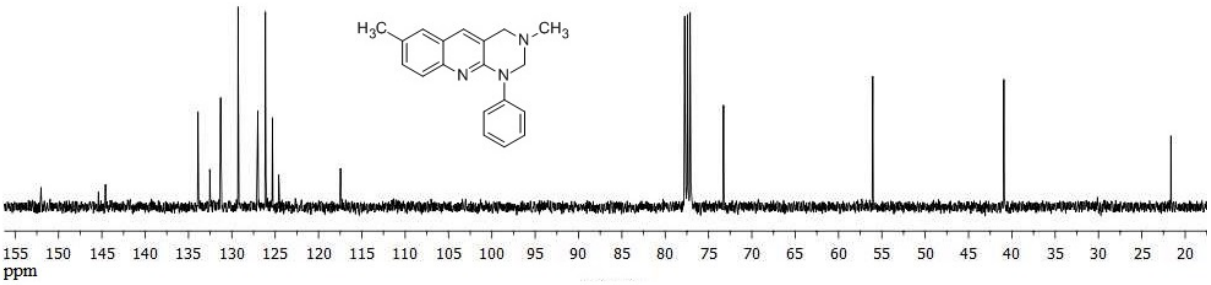

Figure S4. ^{13}C NMR spectrum (100 MHz, $CDCl_3$) of **12**.

Computational Data

Coordinates:

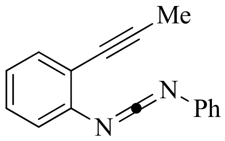

7a

Zero-point correction= 0.225855 (Hartree/Particle)
Thermal correction to Energy= 0.242360
Thermal correction to Enthalpy= 0.243304
Thermal correction to Gibbs Free Energy= 0.178143
Sum of electronic and zero-point Energies= -725.873308
Sum of electronic and thermal Energies= -725.856803
Sum of electronic and thermal Enthalpies= -725.855858
Sum of electronic and thermal Free Energies= -725.921020

C	-3.433438000	-1.713491000	-0.006187000
C	-2.327588000	-0.843160000	-0.135642000
C	-2.512120000	0.570474000	0.033380000
C	-3.818849000	1.047367000	0.326207000
C	-4.907279000	0.171249000	0.447488000
C	-4.713140000	-1.214910000	0.279830000
H	-3.263102000	-2.785169000	-0.137452000
H	-3.958957000	2.123983000	0.455406000
H	-5.555826000	-1.906313000	0.372953000
C	-1.421784000	1.487190000	-0.085060000
C	-0.513026000	2.305302000	-0.184145000
C	0.058698000	-1.025134000	-0.641431000
C	0.586520000	3.264401000	-0.325233000
H	1.007767000	3.544285000	0.657608000
H	1.406903000	2.839629000	-0.930542000
N	-1.086750000	-1.436690000	-0.417005000
N	1.215964000	-0.859662000	-1.050825000
C	2.432380000	-0.636292000	-0.368526000
C	3.602767000	-0.479544000	-1.146501000
C	2.519257000	-0.573838000	1.044706000
C	4.841870000	-0.264191000	-0.519159000
H	3.522681000	-0.533692000	-2.235492000
C	3.762921000	-0.357645000	1.661209000
H	1.614082000	-0.694005000	1.647404000
C	4.929510000	-0.201388000	0.885486000
H	5.741767000	-0.146624000	-1.130873000
H	3.819976000	-0.312089000	2.753471000
H	5.895353000	-0.033996000	1.371255000
H	0.246412000	4.192370000	-0.819606000
H	-5.901748000	0.567743000	0.672132000

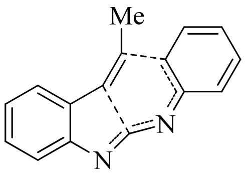

7a_TS$_{DA}$

Zero-point correction= 0.225354 (Hartree/Particle)
Thermal correction to Energy= 0.239847
Thermal correction to Enthalpy= 0.240791
Thermal correction to Gibbs Free Energy= 0.183991
Sum of electronic and zero-point Energies= -725.832160
Sum of electronic and thermal Energies= -725.817667
Sum of electronic and thermal Enthalpies= -725.816723
Sum of electronic and thermal Free Energies= -725.873523

C	-3.844592000	-1.204484000	0.250038000
C	-2.468587000	-0.913014000	0.178971000
C	-2.039293000	0.419932000	-0.124537000
C	-2.966914000	1.443814000	-0.368681000
C	-4.344066000	1.141047000	-0.299110000
C	-4.770517000	-0.170494000	0.006063000
H	-4.173908000	-2.222702000	0.472850000
H	-2.635973000	2.461071000	-0.599774000
H	-5.841872000	-0.389527000	0.054886000
C	-0.567278000	0.452338000	-0.079907000
C	0.370278000	1.328536000	-0.114402000
C	-0.270613000	-1.251266000	0.126560000
C	1.041864000	2.626133000	-0.194138000
H	1.249924000	3.042952000	0.808066000
H	1.998911000	2.577990000	-0.744694000
N	-1.425626000	-1.818024000	0.390142000
N	0.913393000	-1.720855000	-0.155308000
C	2.064225000	-1.002033000	-0.016411000
C	3.128431000	-1.214086000	-0.954568000
C	2.251633000	-0.020089000	1.016434000
C	4.303725000	-0.472526000	-0.870627000
H	2.980611000	-1.965049000	-1.735096000
C	3.469777000	0.698330000	1.103933000
H	1.530040000	0.036517000	1.833329000
C	4.480760000	0.494592000	0.159503000
H	5.103163000	-0.637490000	-1.599788000
H	3.620644000	1.404803000	1.926688000

H	5.419473000	1.052916000	0.226295000
H	0.383014000	3.339932000	-0.729679000
H	-5.081394000	1.930358000	-0.471662000

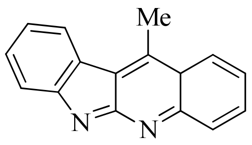

$9a_{DA}$

Zero-point correction= 0.229515 (Hartree/Particle)
Thermal correction to Energy= 0.243123
Thermal correction to Enthalpy= 0.244067
Thermal correction to Gibbs Free Energy= 0.189452
Sum of electronic and zero-point Energies= -725.888700
Sum of electronic and thermal Energies= -725.875092
Sum of electronic and thermal Enthalpies= -725.874148
Sum of electronic and thermal Free Energies= -725.928763

C	-3.756643000	-1.316440000	-0.067689000
C	-2.386955000	-1.006004000	0.001936000
C	-1.932403000	0.360122000	-0.021522000
C	-2.868155000	1.404829000	-0.134327000
C	-4.241832000	1.086821000	-0.202306000
C	-4.679115000	-0.256925000	-0.166914000
H	-4.081793000	-2.360254000	-0.050709000
H	-2.559453000	2.452530000	-0.182907000
H	-5.749956000	-0.475873000	-0.224508000
C	-0.468659000	0.260071000	0.083570000
C	0.568063000	1.141219000	0.196979000
C	-0.219224000	-1.219056000	0.112022000
C	0.423144000	2.644905000	0.249614000
H	-0.608416000	2.948463000	0.474037000
H	1.082268000	3.084529000	1.020528000
N	-1.333709000	-1.940604000	0.084710000
N	1.034818000	-1.790139000	0.119688000
C	2.068674000	-0.970020000	0.154957000
C	3.400757000	-1.526108000	0.001767000
C	1.965099000	0.549535000	0.396288000
C	4.481537000	-0.722297000	-0.270003000
H	3.482339000	-2.616553000	0.010267000
C	3.143139000	1.332664000	-0.166432000
H	2.118389000	0.631942000	1.507691000
C	4.332251000	0.719543000	-0.418374000
H	5.465474000	-1.170245000	-0.439867000
H	3.040240000	2.414805000	-0.281970000
H	5.191653000	1.306769000	-0.757316000

H	0.709444000	3.108443000	-0.714572000
H	-4.976034000	1.893499000	-0.288851000

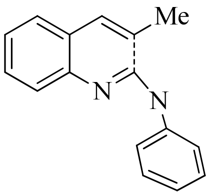

$7a\_TS_{MS}$

Zero-point correction= 0.225251 (Hartree/Particle)
Thermal correction to Energy= 0.239993
Thermal correction to Enthalpy= 0.240937
Thermal correction to Gibbs Free Energy= 0.181927
Sum of electronic and zero-point Energies= -725.835296
Sum of electronic and thermal Energies= -725.820554
Sum of electronic and thermal Enthalpies= -725.819610
Sum of electronic and thermal Free Energies= -725.878619

C	-3.178306000	-1.652199000	-0.520093000
C	-2.232728000	-0.602828000	-0.356139000
C	-2.686863000	0.631303000	0.273924000
C	-4.034665000	0.757198000	0.697278000
C	-4.934281000	-0.297374000	0.515350000
C	-4.501063000	-1.500958000	-0.093501000
H	-2.836073000	-2.578031000	-0.989424000
H	-4.353403000	1.692347000	1.165394000
H	-5.208179000	-2.324191000	-0.233535000
C	-1.658659000	1.585949000	0.379786000
C	-0.407586000	1.664087000	0.047760000
C	0.010870000	0.102239000	-0.669957000
C	0.688804000	2.683520000	0.084049000
H	1.526616000	2.358297000	0.722961000
H	1.094960000	2.847617000	-0.928258000
N	-0.940189000	-0.787166000	-0.780049000
N	1.238806000	0.141623000	-1.106786000
C	2.397477000	-0.159674000	-0.416988000
C	3.643499000	0.066938000	-1.074811000
C	2.410595000	-0.729479000	0.894200000
C	4.849790000	-0.277883000	-0.451754000
H	3.627476000	0.500917000	-2.078301000
C	3.625461000	-1.063020000	1.507286000
H	1.462023000	-0.906217000	1.409465000
C	4.851256000	-0.841735000	0.842265000
H	5.796157000	-0.105470000	-0.973870000
H	3.620009000	-1.500439000	2.510892000

H	5.795579000	-1.104415000	1.328007000
H	0.294776000	3.632915000	0.478349000
H	-5.971863000	-0.191019000	0.844248000

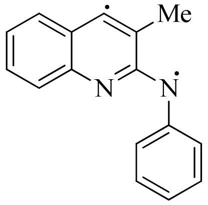

7a_INT$_{MS}$ (Singlet)

Zero-point correction= 0.226791 (Hartree/Particle)
Thermal correction to Energy= 0.241419
Thermal correction to Enthalpy= 0.242363
Thermal correction to Gibbs Free Energy= 0.184335
Sum of electronic and zero-point Energies= -725.842808
Sum of electronic and thermal Energies= -725.828180
Sum of electronic and thermal Enthalpies= -725.827236
Sum of electronic and thermal Free Energies= -725.885264

C	-2.334812000	-1.950713000	-0.336429000
C	-1.816079000	-0.624259000	-0.167447000
C	-2.770430000	0.459705000	0.100296000
C	-4.163456000	0.169769000	0.155299000
C	-4.620358000	-1.136510000	-0.011301000
C	-3.699936000	-2.198913000	-0.251925000
H	-1.617368000	-2.753476000	-0.528052000
H	-4.863388000	0.989689000	0.339630000
H	-4.075408000	-3.219021000	-0.378139000
C	-2.166252000	1.722187000	0.244393000
C	-0.843192000	2.012056000	0.145951000
C	0.025197000	0.839607000	-0.162176000
C	-0.232999000	3.381983000	0.371159000
H	0.442732000	3.376486000	1.244278000
H	0.376129000	3.680855000	-0.498622000
N	-0.479634000	-0.412353000	-0.294574000
N	1.342294000	1.122409000	-0.356429000
C	2.364099000	0.236425000	-0.125452000
C	3.634246000	0.574765000	-0.706594000
C	2.286389000	-0.951040000	0.682436000
C	4.745795000	-0.253337000	-0.537456000
H	3.691416000	1.490933000	-1.300350000
C	3.415354000	-1.757224000	0.860521000
H	1.342971000	-1.206582000	1.164510000
C	4.645340000	-1.425221000	0.248187000
H	5.699282000	0.011401000	-1.004861000

H	3.344711000	-2.653128000	1.485654000
H	5.520881000	-2.064990000	0.394430000
H	-1.021929000	4.131350000	0.539700000
H	-5.692086000	-1.349591000	0.042067000

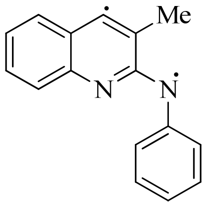

7a_INT$_{MS}$ (Triplet)

Zero-point correction= 0.227053 (Hartree/Particle)
Thermal correction to Energy= 0.241637
Thermal correction to Enthalpy= 0.242581
Thermal correction to Gibbs Free Energy= 0.183397
Sum of electronic and zero-point Energies= -725.841279
Sum of electronic and thermal Energies= -725.826695
Sum of electronic and thermal Enthalpies= -725.825751
Sum of electronic and thermal Free Energies= -725.884935

C	-2.331005000	-1.960741000	-0.290389000
C	-1.823782000	-0.627028000	-0.134805000
C	-2.791121000	0.459845000	0.066445000
C	-4.181665000	0.159445000	0.117772000
C	-4.626767000	-1.153491000	-0.029876000
C	-3.696124000	-2.214851000	-0.236425000
H	-1.605238000	-2.762573000	-0.452172000
H	-4.890643000	0.978077000	0.270751000
H	-4.064139000	-3.238527000	-0.355200000
C	-2.201972000	1.733398000	0.186991000
C	-0.873296000	2.019966000	0.129309000
C	0.014724000	0.838736000	-0.043831000
C	-0.293955000	3.418259000	0.232917000
H	0.348041000	3.515814000	1.125410000
H	0.343347000	3.640315000	-0.639291000
N	-0.482049000	-0.419521000	-0.166169000
N	1.342221000	1.127930000	-0.151061000
C	2.371495000	0.222438000	-0.049710000
C	3.643996000	0.682294000	-0.535403000
C	2.314779000	-1.083125000	0.552092000
C	4.780700000	-0.126571000	-0.466115000
H	3.685208000	1.683679000	-0.972122000
C	3.467964000	-1.870155000	0.636874000
H	1.366487000	-1.444462000	0.946802000
C	4.701685000	-1.409361000	0.122478000

H	5.735241000	0.238658000	-0.857505000
H	3.412248000	-2.856372000	1.109034000
H	5.594145000	-2.038825000	0.191172000
H	-1.101919000	4.163713000	0.294793000
H	-5.697920000	-1.372696000	0.008980000

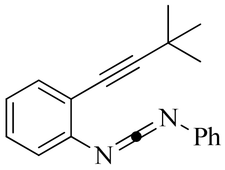

7b

Zero-point correction= 0.308490 (Hartree/Particle)
Thermal correction to Energy= 0.328941
Thermal correction to Enthalpy= 0.329885
Thermal correction to Gibbs Free Energy= 0.256198
Sum of electronic and zero-point Energies= -843.651931
Sum of electronic and thermal Energies= -843.631480
Sum of electronic and thermal Enthalpies= -843.630536
Sum of electronic and thermal Free Energies= -843.704224

C	-3.483608000	-2.342958000	-0.200364000
C	-2.417828000	-1.415557000	-0.251861000
C	-2.650240000	-0.044214000	0.101727000
C	-3.962491000	0.330349000	0.502500000
C	-5.009076000	-0.601569000	0.551005000
C	-4.768462000	-1.944570000	0.196474000
H	-3.276535000	-3.379464000	-0.478831000
H	-4.140280000	1.374653000	0.773046000
H	-5.578461000	-2.679182000	0.228973000
C	-1.614108000	0.940258000	0.055136000
C	-0.781978000	1.843270000	0.028022000
C	-0.021934000	-1.481460000	-0.784310000
C	0.216270000	2.933808000	-0.023914000
N	-1.172171000	-1.916491000	-0.659888000
N	1.143054000	-1.245800000	-1.135450000
C	2.359149000	-1.266631000	-0.416562000
C	3.546851000	-0.973021000	-1.125634000
C	2.429292000	-1.577593000	0.964468000
C	4.786161000	-0.992686000	-0.462975000
H	3.480294000	-0.739326000	-2.191557000
C	3.673316000	-1.594307000	1.616834000
H	1.510894000	-1.803390000	1.514779000
C	4.857113000	-1.302379000	0.909395000
H	5.699202000	-0.766357000	-1.022383000
H	3.716861000	-1.836703000	2.683454000
H	5.823063000	-1.317176000	1.422743000
C	-0.508691000	4.301017000	0.190755000
H	-1.011414000	4.332667000	1.171983000
H	0.226365000	5.124228000	0.150405000
H	-1.266751000	4.473842000	-0.591400000
C	1.280650000	2.726489000	1.101375000
H	2.022407000	3.544107000	1.067587000
H	0.806729000	2.728765000	2.097327000
H	1.812301000	1.770026000	0.973052000
C	0.919496000	2.927930000	-1.419206000
H	1.427775000	1.967561000	-1.601112000
H	0.188535000	3.090138000	-2.228916000
H	1.671314000	3.735941000	-1.461138000
H	-6.007767000	-0.281351000	0.862173000

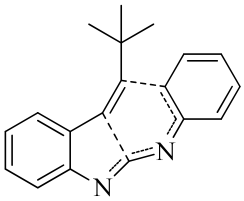

7b_TS$_{DA}$

Zero-point correction= 0.308920 (Hartree/Particle)
Thermal correction to Energy= 0.327046
Thermal correction to Enthalpy= 0.327990
Thermal correction to Gibbs Free Energy= 0.263736
Sum of electronic and zero-point Energies= -843.603096
Sum of electronic and thermal Energies= -843.584969
Sum of electronic and thermal Enthalpies= -843.584025
Sum of electronic and thermal Free Energies= -843.648280

C	-4.067980000	-1.565079000	0.284318000
C	-2.687798000	-1.302841000	0.187729000
C	-2.215074000	0.020499000	-0.101142000
C	-3.131218000	1.058681000	-0.332166000
C	-4.514943000	0.787761000	-0.239553000
C	-4.974913000	-0.509976000	0.068955000
H	-4.410998000	-2.581276000	0.495779000
H	-2.801280000	2.067723000	-0.578638000
H	-6.050632000	-0.701158000	0.134957000
C	-0.726135000	-0.036946000	-0.058776000
C	0.260806000	0.818601000	-0.048170000
C	-0.515561000	-1.672235000	0.069475000
C	0.786094000	2.217844000	-0.081353000
N	-1.673801000	-2.251042000	0.330879000
N	0.629221000	-2.240672000	-0.267852000

C	1.824327000	-1.636947000	-0.083892000
C	2.924033000	-2.021370000	-0.930636000
C	2.051564000	-0.627743000	0.925532000
C	4.183533000	-1.469068000	-0.749731000
H	2.730445000	-2.770924000	-1.702455000
C	3.370883000	-0.115921000	1.115316000
H	1.329264000	-0.521771000	1.736272000
C	4.418017000	-0.517283000	0.290242000
H	5.012220000	-1.777462000	-1.394902000
H	3.567084000	0.567469000	1.947143000
H	5.427684000	-0.127674000	0.451944000
C	-0.350579000	3.161034000	-0.618324000
H	-0.735916000	2.818746000	-1.591833000
H	-1.183128000	3.232585000	0.097128000
H	0.072659000	4.172045000	-0.751982000
C	1.177278000	2.712284000	1.346852000
H	1.483307000	3.771551000	1.294125000
H	0.323390000	2.633170000	2.040098000
H	2.017623000	2.138654000	1.761126000
C	1.999320000	2.333607000	-1.057176000
H	2.830040000	1.686061000	-0.745066000
H	1.703524000	2.048298000	-2.080427000
H	2.358093000	3.377608000	-1.079370000
H	-5.231239000	1.598929000	-0.400103000

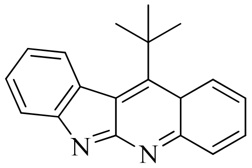

$9b_{DA}$

Zero-point correction= 0.312399 (Hartree/Particle)
Thermal correction to Energy= 0.329937
Thermal correction to Enthalpy= 0.330882
Thermal correction to Gibbs Free Energy= 0.268762
Sum of electronic and zero-point Energies= -843.645102
Sum of electronic and thermal Energies= -843.627563
Sum of electronic and thermal Enthalpies= -843.626619
Sum of electronic and thermal Free Energies= -843.688739

C	-3.838211000	-1.752690000	-0.025233000
C	-2.474912000	-1.431703000	0.098708000
C	-1.988205000	-0.093165000	-0.111647000
C	-2.907080000	0.903763000	-0.498672000
C	-4.274009000	0.578442000	-0.629918000
C	-4.737735000	-0.733240000	-0.386979000
H	-4.170671000	-2.780273000	0.144945000
H	-2.594181000	1.927450000	-0.709423000
H	-5.803265000	-0.959538000	-0.492866000
C	-0.519966000	-0.191673000	0.067132000
C	0.556989000	0.662404000	0.155520000
C	-0.323284000	-1.667640000	0.329297000
C	0.477534000	2.213642000	0.309233000
N	-1.454958000	-2.351540000	0.401142000
N	0.897269000	-2.280196000	0.525178000
C	1.959706000	-1.528371000	0.356681000
C	3.272788000	-2.162352000	0.304647000
C	1.940117000	-0.001473000	0.197099000
C	4.299360000	-1.558206000	-0.377113000
H	3.354231000	-3.182473000	0.690296000
C	2.940531000	0.422409000	-0.889412000
H	2.419615000	0.348180000	1.145037000
C	4.080268000	-0.296487000	-1.079328000
H	5.260488000	-2.069140000	-0.490663000
H	2.751491000	1.330865000	-1.463138000
H	4.829242000	0.042063000	-1.802578000
C	1.801902000	2.857155000	0.835626000
H	2.660698000	2.733433000	0.160187000
H	1.638127000	3.941260000	0.957950000
H	2.084846000	2.459226000	1.825867000
C	-0.601661000	2.589317000	1.377848000
H	-1.600660000	2.200982000	1.151821000
H	-0.310358000	2.196787000	2.367391000
H	-0.673042000	3.688269000	1.458469000
C	0.143196000	2.867228000	-1.072634000
H	-0.015222000	3.953172000	-0.946852000
H	0.970317000	2.730670000	-1.789951000
H	-0.760119000	2.438184000	-1.530118000
H	-4.981688000	1.358867000	-0.925741000

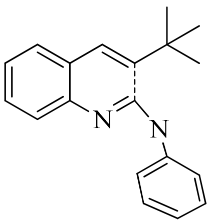

7b_TS_{MS}

Zero-point correction= 0.307547 (Hartree/Particle)
Thermal correction to Energy= 0.326388
Thermal correction to Enthalpy= 0.327332
Thermal correction to Gibbs Free Energy= 0.260139
Sum of electronic and zero-point Energies= -843.607282
Sum of electronic and thermal Energies= -843.588441

Sum of electronic and thermal Enthalpies= -843.587497

Sum of electronic and thermal Free Energies= -843.654690

C	-2.706809000	-2.538208000	-0.256980000
C	-2.031584000	-1.286014000	-0.224583000
C	-2.829639000	-0.090742000	0.046735000
C	-4.235707000	-0.196913000	0.196171000
C	-4.860256000	-1.447215000	0.153543000
C	-4.088371000	-2.616066000	-0.059837000
H	-2.109580000	-3.435117000	-0.439861000
H	-4.814977000	0.713132000	0.373124000
H	-4.578700000	-3.594060000	-0.081849000
C	-2.038732000	1.066290000	0.087433000
C	-0.809836000	1.434721000	-0.048632000
C	0.040689000	-0.139472000	-0.517334000
C	-0.011653000	2.718039000	0.168401000
N	-0.701405000	-1.217707000	-0.527368000
N	1.272076000	0.106529000	-0.815213000
C	2.384815000	-0.578074000	-0.324299000
C	3.648867000	-0.277469000	-0.906621000
C	2.320416000	-1.558555000	0.708071000
C	4.801752000	-0.960446000	-0.494955000
H	3.692529000	0.481414000	-1.692624000
C	3.483038000	-2.230133000	1.114707000
H	1.359444000	-1.784950000	1.176007000
C	4.727962000	-1.939588000	0.518307000
H	5.763706000	-0.727465000	-0.962407000
H	3.419159000	-2.983658000	1.906454000
H	5.630633000	-2.464925000	0.844199000
C	-1.018586000	3.808925000	0.655803000
H	-0.482142000	4.760927000	0.814151000
H	-1.812578000	3.977506000	-0.090229000
H	-1.492832000	3.514742000	1.607036000
C	1.077391000	2.529477000	1.266277000
H	0.626596000	2.195765000	2.216694000
H	1.842464000	1.800970000	0.965719000
H	1.579740000	3.496199000	1.446644000
C	0.636103000	3.196897000	-1.165790000
H	-0.137755000	3.393206000	-1.927618000
H	1.188416000	4.136344000	-0.986164000
H	1.334526000	2.446203000	-1.561236000
H	-5.942519000	-1.520715000	0.291537000

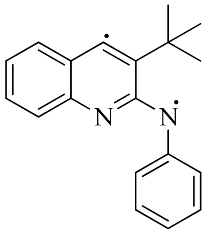

7b_INT$_{MS}$ (Singlet)

Zero-point correction= 0.308871 (Hartree/Particle)

Thermal correction to Energy= 0.327570

Thermal correction to Enthalpy= 0.328515

Thermal correction to Gibbs Free Energy= 0.262005

Sum of electronic and zero-point Energies= -843.615291

Sum of electronic and thermal Energies= -843.596592

Sum of electronic and thermal Enthalpies= -843.595648

Sum of electronic and thermal Free Energies= -843.662157

C	-2.074906000	-2.782576000	-0.250453000
C	-1.669947000	-1.409284000	-0.169760000
C	-2.709387000	-0.393932000	0.017076000
C	-4.076269000	-0.788344000	0.078315000
C	-4.423823000	-2.135859000	-0.003029000
C	-3.417123000	-3.133889000	-0.160799000
H	-1.292364000	-3.535369000	-0.380361000
H	-4.842145000	-0.017150000	0.200627000
H	-3.707836000	-4.187292000	-0.219276000
C	-2.200596000	0.913956000	0.086506000
C	-0.910524000	1.328351000	-0.019238000
C	0.051184000	0.203818000	-0.259522000
C	-0.491217000	2.812937000	0.143251000
N	-0.356951000	-1.090541000	-0.305397000
N	1.353584000	0.528353000	-0.494590000
C	2.410845000	-0.292676000	-0.189434000
C	3.654687000	0.008526000	-0.842442000
C	2.393035000	-1.370211000	0.762375000
C	4.796796000	-0.758933000	-0.603220000
H	3.669110000	0.843032000	-1.548576000
C	3.552232000	-2.113189000	1.009110000
H	1.471926000	-1.589397000	1.302230000
C	4.754929000	-1.825080000	0.325109000
H	5.728971000	-0.526798000	-1.127509000
H	3.526501000	-2.923848000	1.744368000
H	5.654604000	-2.414819000	0.525174000
C	-1.738356000	3.660734000	0.529587000
H	-2.173683000	3.326270000	1.487109000
H	-1.449242000	4.721016000	0.637780000

H	-2.524129000	3.598468000	-0.242660000
C	0.572092000	2.971121000	1.271757000
H	0.186281000	2.593259000	2.235246000
H	1.500979000	2.433469000	1.030835000
H	0.817485000	4.040586000	1.401103000
C	0.075914000	3.355557000	-1.206191000
H	0.966012000	2.792467000	-1.520777000
H	-0.683723000	3.287868000	-2.004585000
H	0.352919000	4.419079000	-1.089471000
H	-5.475503000	-2.431686000	0.054667000

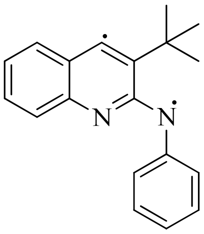

7b_INT$_{MS}$ (Triplet)

Zero-point correction= 0.309143 (Hartree/Particle)
Thermal correction to Energy= 0.327796
Thermal correction to Enthalpy= 0.328741
Thermal correction to Gibbs Free Energy= 0.261247
Sum of electronic and zero-point Energies= -843.613440
Sum of electronic and thermal Energies= -843.594787
Sum of electronic and thermal Enthalpies= -843.593842
Sum of electronic and thermal Free Energies= -843.661335

C	-1.998113000	-2.816765000	-0.299577000
C	-1.631794000	-1.435467000	-0.170231000
C	-2.698684000	-0.453073000	0.031918000
C	-4.050773000	-0.890600000	0.115017000
C	-4.360495000	-2.244846000	-0.005809000
C	-3.329270000	-3.208170000	-0.215910000
H	-1.196112000	-3.541768000	-0.464244000
H	-4.837357000	-0.146496000	0.269806000
H	-3.592592000	-4.265812000	-0.313808000
C	-2.233744000	0.872208000	0.120178000
C	-0.948848000	1.316823000	0.032271000
C	0.050467000	0.219557000	-0.132518000
C	-0.591666000	2.825152000	0.104855000
N	-0.321466000	-1.084530000	-0.223188000
N	1.358835000	0.583248000	-0.283436000
C	2.431088000	-0.257725000	-0.104352000
C	3.664130000	0.165311000	-0.709314000
C	2.451799000	-1.452758000	0.695939000
C	4.833518000	-0.585809000	-0.568255000
H	3.650676000	1.086201000	-1.298368000
C	3.637842000	-2.177442000	0.851465000
H	1.537719000	-1.776448000	1.191956000
C	4.830006000	-1.762764000	0.214800000
H	5.756186000	-0.254709000	-1.054865000
H	3.640956000	-3.076632000	1.475991000
H	5.749356000	-2.342850000	0.339774000
C	-1.887677000	3.651228000	0.354053000
H	-2.370732000	3.369420000	1.305600000
H	-1.639779000	4.726261000	0.402154000
H	-2.622222000	3.507365000	-0.456852000
C	0.395275000	3.102912000	1.280375000
H	-0.036205000	2.777025000	2.243492000
H	1.353162000	2.583418000	1.131810000
H	0.593690000	4.187844000	1.348300000
C	0.036100000	3.295765000	-1.244567000
H	0.966379000	2.752979000	-1.463043000
H	-0.668602000	3.138346000	-2.080054000
H	0.261376000	4.376267000	-1.189705000
H	-5.402446000	-2.572655000	0.056406000

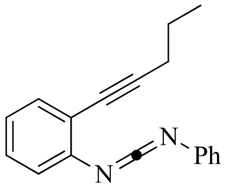

7c

Zero-point correction= 0.281649 (Hartree/Particle)
Thermal correction to Energy= 0.300739
Thermal correction to Enthalpy= 0.301683
Thermal correction to Gibbs Free Energy= 0.229488
Sum of electronic and zero-point Energies= -804.392317
Sum of electronic and thermal Energies= -804.373227
Sum of electronic and thermal Enthalpies= -804.372283
Sum of electronic and thermal Free Energies= -804.444478

C	-3.832797000	-1.759923000	-0.385292000
C	-2.622764000	-1.036951000	-0.283962000
C	-2.637018000	0.305929000	0.223613000
C	-3.886291000	0.865083000	0.608025000
C	-5.079567000	0.136614000	0.497059000
C	-5.052111000	-1.181310000	-0.002480000
H	-3.791249000	-2.781390000	-0.772136000
H	-3.896987000	1.887473000	0.995383000
H	-5.977499000	-1.757908000	-0.092171000

C	-1.437459000	1.072989000	0.349545000
C	-0.430222000	1.762878000	0.478925000
C	-0.259003000	-1.403372000	-0.798096000
C	0.777007000	2.592823000	0.585043000
H	0.778382000	3.119375000	1.559927000
H	1.670203000	1.939163000	0.587637000
N	-1.453970000	-1.703942000	-0.684024000
N	0.924155000	-1.300973000	-1.150371000
C	2.133022000	-1.387308000	-0.425239000
C	3.341419000	-1.221499000	-1.140849000
C	2.175679000	-1.638356000	0.969056000
C	4.574119000	-1.307273000	-0.471201000
H	3.294786000	-1.031425000	-2.216455000
C	3.413410000	-1.721189000	1.628393000
H	1.241342000	-1.765141000	1.523966000
C	4.617860000	-1.556356000	0.914696000
H	5.503269000	-1.180033000	-1.035418000
H	3.435882000	-1.916372000	2.705309000
H	5.578747000	-1.622843000	1.433463000
C	0.914031000	3.642345000	-0.561599000
H	0.019705000	4.291121000	-0.560815000
H	0.918804000	3.111327000	-1.529953000
C	2.190441000	4.498715000	-0.419642000
H	3.099766000	3.871598000	-0.444060000
H	2.266184000	5.231599000	-1.241467000
H	2.194828000	5.060457000	0.532116000
H	-6.025980000	0.594362000	0.799472000

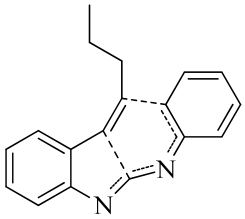

7c_TS$_{DA}$

Zero-point correction= 0.281293 (Hartree/Particle)
Thermal correction to Energy= 0.298453
Thermal correction to Enthalpy= 0.299397
Thermal correction to Gibbs Free Energy= 0.235818
Sum of electronic and zero-point Energies= -804.350731
Sum of electronic and thermal Energies= -804.333572
Sum of electronic and thermal Enthalpies= -804.332628
Sum of electronic and thermal Free Energies= -804.396207

C	-4.023066000	-1.480678000	0.128977000
C	-2.630222000	-1.275273000	0.098238000
C	-2.106053000	0.051895000	-0.035567000
C	-2.963033000	1.156370000	-0.155549000
C	-4.358354000	0.941008000	-0.126858000
C	-4.876157000	-0.365119000	0.013391000
H	-4.420340000	-2.494824000	0.221819000
H	-2.565941000	2.169401000	-0.262337000
H	-5.960263000	-0.515330000	0.032174000
C	-0.632739000	-0.034900000	0.020105000
C	0.372818000	0.764765000	0.120447000
C	-0.462411000	-1.747253000	0.004427000
C	1.136216000	2.015375000	0.216503000
H	1.428320000	2.194395000	1.269754000
H	2.083623000	1.931305000	-0.350837000
N	-1.654335000	-2.270719000	0.190342000
N	0.686011000	-2.272493000	-0.338585000
C	1.882936000	-1.667901000	-0.104305000
C	2.948750000	-1.854169000	-1.047998000
C	2.121366000	-0.823510000	1.036124000
C	4.173311000	-1.219785000	-0.865893000
H	2.760750000	-2.499102000	-1.910427000
C	3.391160000	-0.217397000	1.219762000
H	1.400630000	-0.822124000	1.855637000
C	4.401749000	-0.390794000	0.271078000
H	4.973505000	-1.362462000	-1.598978000
H	3.579054000	0.376005000	2.120509000
H	5.378943000	0.080966000	0.412324000
C	0.339454000	3.255476000	-0.313436000
H	0.010682000	3.057465000	-1.348513000
H	-0.570597000	3.388929000	0.296205000
C	1.196804000	4.539693000	-0.267145000
H	0.620381000	5.404010000	-0.639231000
H	1.521927000	4.770363000	0.763022000
H	2.100737000	4.442154000	-0.894086000
H	-5.038312000	1.794519000	-0.203123000

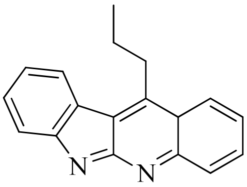

9c$_{DA}$

Zero-point correction= 0.285255 (Hartree/Particle)
Thermal correction to Energy= 0.301558
Thermal correction to Enthalpy= 0.302502
Thermal correction to Gibbs Free Energy= 0.241328
Sum of electronic and zero-point Energies= -804.405830
Sum of electronic and thermal Energies= -804.389527

Sum of electronic and thermal Enthalpies= -804.388583

Sum of electronic and thermal Free Energies= -804.449757

C	-3.837984000	-1.686070000	-0.199564000
C	-2.459236000	-1.430869000	-0.093758000
C	-1.965471000	-0.091649000	0.096832000
C	-2.869410000	0.984073000	0.168352000
C	-4.252475000	0.720599000	0.065265000
C	-4.728710000	-0.598049000	-0.116090000
H	-4.195004000	-2.709023000	-0.346983000
H	-2.527252000	2.014947000	0.293413000
H	-5.805901000	-0.774113000	-0.197777000
C	-0.506297000	-0.247157000	0.176476000
C	0.550089000	0.585450000	0.417349000
C	-0.298497000	-1.717612000	-0.024263000
C	0.414237000	2.075575000	0.667770000
H	-0.523556000	2.270464000	1.216957000
H	1.232234000	2.426448000	1.324432000
N	-1.432538000	-2.395436000	-0.164632000
N	0.940060000	-2.315433000	-0.103712000
C	1.994461000	-1.540062000	0.071804000
C	3.311712000	-2.108095000	-0.150043000
C	1.927824000	-0.073071000	0.548146000
C	4.424331000	-1.310470000	-0.264078000
H	3.357626000	-3.187562000	-0.318702000
C	3.149597000	0.744913000	0.155135000
H	2.047047000	-0.174154000	1.662341000
C	4.325440000	0.139614000	-0.168050000
H	5.397178000	-1.759022000	-0.487676000
H	3.091929000	1.833604000	0.227465000
H	5.213251000	0.744323000	-0.378734000
C	0.414461000	2.928546000	-0.640666000
H	1.334628000	2.721948000	-1.216775000
H	-0.426230000	2.601307000	-1.277574000
C	0.304126000	4.442869000	-0.358091000
H	0.293697000	5.019448000	-1.299149000
H	-0.623143000	4.682444000	0.192766000
H	1.155146000	4.803406000	0.247650000
H	-4.963502000	1.550425000	0.121613000

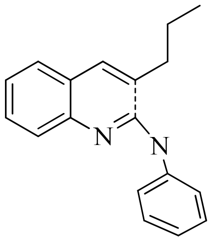

7c_TS$_{MS}$

Zero-point correction= 0.280594 (Hartree/Particle)

Thermal correction to Energy= 0.298108

Thermal correction to Enthalpy= 0.299052

Thermal correction to Gibbs Free Energy= 0.233081

Sum of electronic and zero-point Energies= -804.354363

Sum of electronic and thermal Energies= -804.336849

Sum of electronic and thermal Enthalpies= -804.335905

Sum of electronic and thermal Free Energies= -804.401876

C	-3.461027000	-1.965481000	-0.368503000
C	-2.424114000	-0.992622000	-0.312196000
C	-2.762258000	0.333223000	0.195804000
C	-4.087999000	0.616347000	0.614495000
C	-5.077644000	-0.367608000	0.540347000
C	-4.759098000	-1.657838000	0.047667000
H	-3.206760000	-2.958735000	-0.747221000
H	-4.317829000	1.616701000	0.991023000
H	-5.537411000	-2.424937000	-0.008636000
C	-1.651816000	1.194713000	0.199314000
C	-0.400643000	1.113311000	-0.149217000
C	-0.133329000	-0.514120000	-0.691431000
C	0.769297000	2.055240000	-0.229641000
H	1.591872000	1.679453000	0.406312000
H	1.162353000	2.028429000	-1.263539000
N	-1.158047000	-1.331207000	-0.714137000
N	1.088389000	-0.651053000	-1.139885000
C	2.225889000	-0.949621000	-0.419807000
C	3.478403000	-0.916210000	-1.105406000
C	2.212318000	-1.339488000	0.956869000
C	4.660993000	-1.273027000	-0.445538000
H	3.483327000	-0.619909000	-2.157921000
C	3.404472000	-1.686851000	1.605120000
H	1.259811000	-1.368277000	1.493947000
C	4.634907000	-1.657616000	0.912587000
H	5.610973000	-1.250105000	-0.988677000
H	3.377902000	-1.985134000	2.658206000
H	5.561528000	-1.930286000	1.426131000
C	0.420727000	3.509804000	0.177541000
H	-0.406896000	3.873837000	-0.457958000
H	0.044556000	3.516726000	1.216353000
C	1.636245000	4.454054000	0.052680000
H	1.367373000	5.483172000	0.347986000
H	2.469538000	4.126622000	0.700360000
H	2.012818000	4.490985000	-0.985495000
H	-6.097486000	-0.139981000	0.863183000

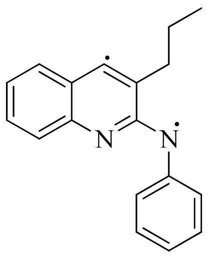

7c_INT$_{MS}$ (Singlet)

Zero-point correction= 0.282133 (Hartree/Particle)
Thermal correction to Energy= 0.299510
Thermal correction to Enthalpy= 0.300455
Thermal correction to Gibbs Free Energy= 0.235288
Sum of electronic and zero-point Energies= -804.359276
Sum of electronic and thermal Energies= -804.341899
Sum of electronic and thermal Enthalpies= -804.340954
Sum of electronic and thermal Free Energies= -804.406121

C	-1.586888000	-2.883571000	-0.402217000
C	-1.352389000	-1.487336000	-0.173432000
C	-2.504159000	-0.639826000	0.159151000
C	-3.805150000	-1.216600000	0.217692000
C	-3.983917000	-2.580295000	-0.008349000
C	-2.868366000	-3.414883000	-0.313569000
H	-0.722528000	-3.509007000	-0.642204000
H	-4.656021000	-0.570597000	0.452017000
H	-3.026372000	-4.483908000	-0.486074000
C	-2.172107000	0.711987000	0.358745000
C	-0.944259000	1.283024000	0.257196000
C	0.143750000	0.328133000	-0.115410000
C	-0.608901000	2.738211000	0.564640000
H	-0.075358000	2.766874000	1.535330000
H	0.131510000	3.084387000	-0.178368000
N	-0.093273000	-0.993820000	-0.304219000
N	1.373864000	0.878724000	-0.307358000
C	2.555477000	0.208749000	-0.116246000
C	3.723520000	0.810554000	-0.698503000
C	2.729052000	-0.992952000	0.654857000
C	4.980378000	0.216723000	-0.565721000
H	3.587914000	1.736204000	-1.264185000
C	3.998773000	-1.561872000	0.797499000
H	1.863400000	-1.445525000	1.138231000
C	5.128164000	-0.973481000	0.184146000
H	5.854449000	0.679940000	-1.033865000
H	4.117898000	-2.471164000	1.395367000
H	6.115943000	-1.429150000	0.302106000
C	-1.818465000	3.705990000	0.617088000
H	-2.573339000	3.317249000	1.325715000
H	-1.461026000	4.664930000	1.036530000
C	-2.479016000	3.972084000	-0.754748000
H	-2.881974000	3.045461000	-1.197721000
H	-3.314037000	4.688303000	-0.657784000
H	-1.751214000	4.398726000	-1.468782000
H	-4.986356000	-3.014879000	0.047804000

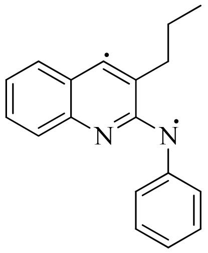

7c_INT$_{MS}$ (Triplet)

Zero-point correction= 0.282345 (Hartree/Particle)
Thermal correction to Energy= 0.299701
Thermal correction to Enthalpy= 0.300645
Thermal correction to Gibbs Free Energy= 0.234140
Sum of electronic and zero-point Energies= -804.357772
Sum of electronic and thermal Energies= -804.340416
Sum of electronic and thermal Enthalpies= -804.339471
Sum of electronic and thermal Free Energies= -804.405977

C	1.563912000	-2.875306000	0.409705000
C	1.345549000	-1.481381000	0.147072000
C	2.511355000	-0.646806000	-0.169033000
C	3.804043000	-1.241616000	-0.223770000
C	3.966556000	-2.602688000	0.030240000
C	2.841210000	-3.419447000	0.349828000
H	0.691561000	-3.486829000	0.656507000
H	4.663677000	-0.609488000	-0.464037000
H	2.988704000	-4.484919000	0.551066000
C	2.200874000	0.708223000	-0.389959000
C	0.969351000	1.283819000	-0.326893000
C	-0.141275000	0.333432000	-0.035910000
C	0.677079000	2.761161000	-0.567155000
H	0.187951000	2.853627000	-1.556870000
H	-0.088887000	3.085948000	0.158641000
N	0.081274000	-0.987670000	0.185359000
N	-1.378377000	0.896324000	0.077239000
C	-2.569161000	0.212317000	0.031814000
C	-3.710306000	0.921192000	0.542988000
C	-2.788545000	-1.088113000	-0.542843000

C	-4.984509000	0.349087000	0.525222000
H	-3.541124000	1.918218000	0.958377000
C	-4.075180000	-1.635405000	-0.577266000
H	-1.943287000	-1.634090000	-0.959457000
C	-5.177235000	-0.933655000	-0.036867000
H	-5.835936000	0.900355000	0.936228000
H	-4.228890000	-2.620339000	-1.029930000
H	-6.177348000	-1.376757000	-0.065641000
C	1.908085000	3.701043000	-0.514044000
H	2.686021000	3.331748000	-1.208137000
H	1.591579000	4.689364000	-0.896459000
C	2.511488000	3.877751000	0.898001000
H	2.873998000	2.919317000	1.307389000
H	3.365682000	4.577464000	0.877365000
H	1.761662000	4.283049000	1.601672000
H	4.964619000	-3.048888000	-0.011665000

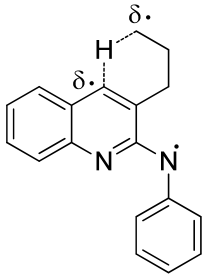

7c_TS$_{\gamma HT}$

Zero-point correction= 0.277372 (Hartree/Particle)
Thermal correction to Energy= 0.293730
Thermal correction to Enthalpy= 0.294674
Thermal correction to Gibbs Free Energy= 0.232818
Sum of electronic and zero-point Energies= -804.347140
Sum of electronic and thermal Energies= -804.330783
Sum of electronic and thermal Enthalpies= -804.329838
Sum of electronic and thermal Free Energies= -804.391694

C	-1.555754000	-2.837653000	-0.160320000
C	-1.303228000	-1.423066000	-0.140722000
C	-2.476203000	-0.535900000	-0.041348000
C	-3.788208000	-1.089035000	-0.047203000
C	-3.978073000	-2.467793000	-0.071647000
C	-2.850130000	-3.340415000	-0.115814000
H	-0.687823000	-3.501328000	-0.199422000
H	-4.645466000	-0.409142000	-0.004577000
H	-3.008363000	-4.423500000	-0.121668000
C	-2.133031000	0.829252000	-0.021243000
C	-0.878655000	1.345946000	-0.073214000
C	0.241833000	0.365781000	-0.296259000
C	-0.617497000	2.811351000	0.216679000
H	-0.310457000	2.929805000	1.275157000
H	0.234633000	3.161998000	-0.387411000
N	-0.040034000	-0.969497000	-0.303238000
N	1.455729000	0.897801000	-0.462549000
C	2.642416000	0.255951000	-0.160576000
C	3.831649000	0.834091000	-0.711930000
C	2.787486000	-0.892932000	0.684530000
C	5.087945000	0.266081000	-0.476633000
H	3.721088000	1.723076000	-1.339269000
C	4.055052000	-1.439630000	0.927708000
H	1.901883000	-1.333423000	1.143006000
C	5.211106000	-0.875615000	0.346924000
H	5.979628000	0.715398000	-0.925845000
H	4.145898000	-2.314529000	1.580220000
H	6.195655000	-1.311110000	0.543257000
C	-3.128873000	3.107376000	0.618714000
H	-3.118856000	3.072351000	1.717969000
H	-4.106790000	3.380205000	0.198470000
H	-2.985942000	1.793283000	0.254465000
C	-1.892832000	3.665305000	-0.057100000
H	-2.065173000	3.730789000	-1.146296000
H	-1.700141000	4.696651000	0.301447000
H	-4.989647000	-2.883221000	-0.052263000

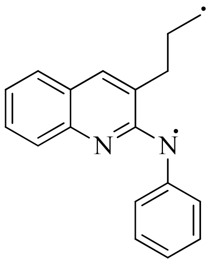

7c_INT$_{\gamma HT}$ (Singlet)

Zero-point correction= 0.280403 (Hartree/Particle)
Thermal correction to Energy= 0.297922
Thermal correction to Enthalpy= 0.298866
Thermal correction to Gibbs Free Energy= 0.233838
Sum of electronic and zero-point Energies= -804.372003
Sum of electronic and thermal Energies= -804.354485
Sum of electronic and thermal Enthalpies= -804.353541
Sum of electronic and thermal Free Energies= -804.418568

C	-1.520493000	-2.907590000	-0.197564000
C	-1.321921000	-1.486814000	-0.166921000
C	-2.479544000	-0.621723000	-0.064722000

C	-3.777550000	-1.202418000	0.014778000
C	-3.938161000	-2.587340000	-0.007378000
C	-2.800632000	-3.442691000	-0.114831000
H	-0.636035000	-3.544803000	-0.283998000
H	-4.649980000	-0.544137000	0.091881000
H	-2.942167000	-4.527756000	-0.134907000
C	-2.244255000	0.790039000	-0.064354000
C	-0.963551000	1.305755000	-0.159334000
C	0.147216000	0.344263000	-0.231738000
C	-0.651587000	2.796995000	-0.184682000
H	-0.020499000	3.047799000	0.689558000
H	-0.001114000	2.990963000	-1.056126000
N	-0.049644000	-1.000998000	-0.235432000
N	1.396804000	0.884570000	-0.377631000
C	2.571553000	0.221928000	-0.119491000
C	3.752386000	0.798398000	-0.702539000
C	2.737713000	-0.925259000	0.733179000
C	5.013142000	0.235557000	-0.490728000
H	3.624372000	1.683951000	-1.330941000
C	4.010652000	-1.460195000	0.957395000
H	1.862381000	-1.362338000	1.211680000
C	5.152687000	-0.898078000	0.342650000
H	5.895405000	0.680995000	-0.961021000
H	4.121629000	-2.325357000	1.619189000
H	6.141859000	-1.330085000	0.522609000
C	-2.646437000	3.849730000	1.058380000
H	-2.174905000	3.628550000	2.020711000
H	-3.654854000	4.273408000	1.074086000
H	-3.105026000	1.460747000	0.017019000
C	-1.865974000	3.760744000	-0.218764000
H	-2.536433000	3.508426000	-1.065485000
H	-1.466129000	4.769815000	-0.476815000
H	-4.940069000	-3.022991000	0.053985000

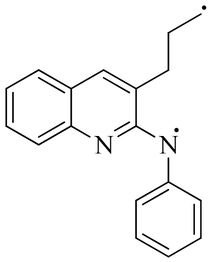

7c_INT$_{\gamma HT}$ (Triplet)

Zero-point correction= 0.280387 (Hartree/Particle)
Thermal correction to Energy= 0.297938
Thermal correction to Enthalpy= 0.298883
Thermal correction to Gibbs Free Energy= 0.232743
Sum of electronic and zero-point Energies= -804.371662
Sum of electronic and thermal Energies= -804.354111
Sum of electronic and thermal Enthalpies= -804.353167
Sum of electronic and thermal Free Energies= -804.419306

C	-1.522172000	-2.907688000	-0.199424000
C	-1.323593000	-1.487254000	-0.170502000
C	-2.479706000	-0.620897000	-0.066849000
C	-3.777490000	-1.200618000	0.016221000
C	-3.938822000	-2.585897000	-0.004826000
C	-2.802658000	-3.442238000	-0.114073000
H	-0.638080000	-3.545168000	-0.287569000
H	-4.649459000	-0.542019000	0.095132000
H	-2.945035000	-4.527172000	-0.133441000
C	-2.243118000	0.791390000	-0.066171000
C	-0.962523000	1.305545000	-0.164684000
C	0.145311000	0.343163000	-0.239268000
C	-0.648084000	2.796850000	-0.193207000
H	-0.003571000	3.044146000	0.672105000
H	-0.009530000	2.989363000	-1.073864000
N	-0.050523000	-1.001769000	-0.238492000
N	1.396956000	0.878721000	-0.399773000
C	2.569326000	0.218809000	-0.128817000
C	3.751615000	0.774148000	-0.729589000
C	2.731611000	-0.905897000	0.754422000
C	5.009978000	0.211317000	-0.504589000
H	3.626310000	1.642908000	-1.381499000
C	4.002200000	-1.440411000	0.990899000
H	1.855134000	-1.325476000	1.246570000
C	5.145639000	-0.900083000	0.358973000
H	5.893559000	0.639597000	-0.988094000
H	4.110721000	-2.287703000	1.675767000
H	6.133155000	-1.331551000	0.548935000
C	-2.629388000	3.855144000	1.073596000
H	-2.144458000	3.651298000	2.033193000
H	-3.642086000	4.268067000	1.095577000
H	-3.102955000	1.462490000	0.018056000
C	-1.858090000	3.768499000	-0.210794000
H	-2.536415000	3.524532000	-1.053397000
H	-1.451570000	4.775380000	-0.466636000
H	-4.940969000	-3.020650000	0.059112000

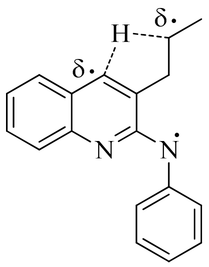

7c_TS$_{\beta HT}$

Zero-point correction= 0.276845 (Hartree/Particle)
Thermal correction to Energy= 0.293767
Thermal correction to Enthalpy= 0.294712
Thermal correction to Gibbs Free Energy= 0.230944
Sum of electronic and zero-point Energies= -804.338840
Sum of electronic and thermal Energies= -804.321918
Sum of electronic and thermal Enthalpies= -804.320974
Sum of electronic and thermal Free Energies= -804.384741

C	1.339949000	-2.966346000	-0.287292000
C	1.199893000	-1.544313000	-0.150026000
C	2.414915000	-0.749213000	0.051549000
C	3.680507000	-1.400302000	0.086493000
C	3.768924000	-2.785116000	-0.047135000
C	2.591096000	-3.569017000	-0.230997000
H	0.428373000	-3.552766000	-0.433255000
H	4.580991000	-0.793839000	0.225544000
H	2.677504000	-4.655210000	-0.333525000
C	2.187457000	0.644083000	0.167426000
C	0.942586000	1.219073000	0.086219000
C	-0.206890000	0.347576000	-0.132893000
C	0.950589000	2.730182000	0.283451000
H	0.592744000	3.243158000	-0.629165000
H	0.264350000	3.036879000	1.092835000
N	-0.048746000	-1.002048000	-0.237044000
N	-1.414919000	0.970319000	-0.280510000
C	-2.635085000	0.364797000	-0.101622000
C	-3.764844000	1.078410000	-0.631813000
C	-2.890215000	-0.857102000	0.614019000
C	-5.062531000	0.578466000	-0.501416000
H	-3.567447000	2.018060000	-1.154821000
C	-4.198520000	-1.331520000	0.756839000
H	-2.053581000	-1.401152000	1.050473000
C	-5.290144000	-0.630661000	0.195412000
H	-5.905261000	1.130175000	-0.929711000
H	-4.377143000	-2.257506000	1.313151000
H	-6.307976000	-1.015328000	0.311752000

C	3.152971000	4.053940000	-0.319151000
H	2.714351000	5.071380000	-0.255762000
H	4.222923000	4.142818000	-0.061282000
H	3.076307000	3.731773000	-1.373119000
C	2.426500000	3.093464000	0.609650000
H	2.901223000	1.851692000	0.440963000
H	2.608973000	3.289253000	1.678903000
H	4.746377000	-3.275695000	-0.012381000

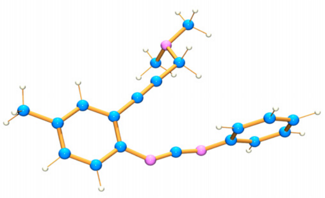

11

Zero-point correction= 0.324184 (Hartree/Particle)
Thermal correction to Energy= 0.346265
Thermal correction to Enthalpy= 0.347209
Thermal correction to Gibbs Free Energy= 0.267869
Sum of electronic and zero-point Energies= -898.956306
Sum of electronic and thermal Energies= -898.934225
Sum of electronic and thermal Enthalpies= -898.933281
Sum of electronic and thermal Free Energies= -899.012621

C	-3.312500	-2.247700	-0.492600
C	-2.150200	-1.450300	-0.390800
C	-2.265800	-0.111100	0.104800
C	-3.553200	0.365900	0.475700
C	-4.710200	-0.425400	0.367700
C	-4.567100	-1.745400	-0.124800
H	-3.206500	-3.268400	-0.869400
H	-3.627600	1.389200	0.855500
H	-5.449200	-2.387800	-0.218800
C	-1.127200	0.743600	0.231900
C	-0.181800	1.515200	0.356100
C	0.241700	-1.673400	-0.855900
C	0.979800	2.407200	0.491600
H	1.286600	2.445300	1.553400
H	1.841900	1.966500	-0.069400
N	-0.937000	-2.041600	-0.777500
N	1.426200	-1.493500	-1.171800
C	2.616900	-1.548400	-0.413200
C	3.835900	-1.299900	-1.085300
C	2.631300	-1.847400	0.972100
C	5.051300	-1.351200	-0.381500

H	3.811200	-1.073000	-2.154400
C	3.851900	-1.895500	1.665900
H	1.688500	-2.038300	1.493400
C	5.067000	-1.648100	0.995500
H	5.989100	-1.159300	-0.912200
H	3.852700	-2.128200	2.735500
H	6.014600	-1.687500	1.540800
C	-6.075400	0.115700	0.766500
H	-6.004200	1.147200	1.148600
H	-6.772000	0.122600	-0.091700
H	-6.539900	-0.504200	1.554800
N	0.704200	3.802500	0.060700
C	0.408000	3.885000	-1.381400
H	0.154400	4.926600	-1.638300
H	1.269000	3.565000	-2.017500
H	-0.456400	3.246400	-1.619800
C	1.830800	4.679000	0.423700
H	2.786300	4.397700	-0.083400
H	1.586400	5.716700	0.143500
H	1.993500	4.645500	1.514200

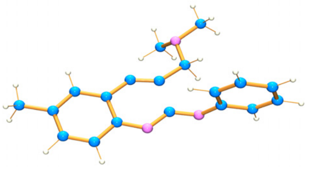

11_TS$_{MS}$

Zero-point correction= 0.323094 (Hartree/Particle)
Thermal correction to Energy= 0.343690
Thermal correction to Enthalpy= 0.344635
Thermal correction to Gibbs Free Energy= 0.271693
Sum of electronic and zero-point Energies= -898.918004
Sum of electronic and thermal Energies= -898.897408
Sum of electronic and thermal Enthalpies= -898.896463
Sum of electronic and thermal Free Energies= -898.969405

C	-3.131300	-2.101600	-0.614300
C	-2.076000	-1.158700	-0.473300
C	-2.407700	0.131800	0.113700
C	-3.736400	0.407300	0.528700
C	-4.759300	-0.537700	0.375700
C	-4.430200	-1.795100	-0.205200
H	-2.892500	-3.074200	-1.052300
H	-3.949800	1.384900	0.971000
H	-5.217800	-2.545700	-0.333100
C	-1.284600	0.972600	0.197100
C	-0.031900	0.909900	-0.119400
C	0.241500	-0.722700	-0.752400
C	1.170400	1.821700	-0.175700
H	1.966500	1.401800	0.461300
H	1.567600	1.794500	-1.219800
N	-0.803500	-1.499700	-0.856700
N	1.474300	-0.846300	-1.161500
C	2.592000	-1.150200	-0.405700
C	3.865500	-1.093600	-1.046600
C	2.533800	-1.566000	0.960800
C	5.028600	-1.451900	-0.352300
H	3.904500	-0.778100	-2.092800
C	3.706300	-1.915400	1.644300
H	1.563600	-1.613500	1.464000
C	4.959600	-1.862100	0.996500
H	5.996300	-1.410500	-0.862200
H	3.645000	-2.233900	2.690000
H	5.870300	-2.136600	1.536700
C	-6.184700	-0.239300	0.815000
H	-6.269500	0.769900	1.250400
H	-6.889200	-0.299500	-0.034700
H	-6.532300	-0.963600	1.574200
N	0.896900	3.191000	0.291500
C	0.026400	3.943500	-0.626900
H	-0.164000	4.944500	-0.206300
H	0.475100	4.067500	-1.642400
H	-0.940200	3.426600	-0.735600
C	2.157000	3.912200	0.546800
H	2.778300	4.049400	-0.371500
H	1.925300	4.909300	0.955800
H	2.756100	3.364400	1.292900

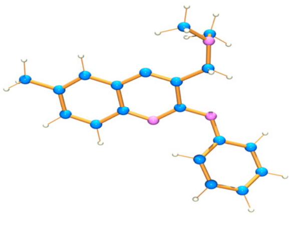

11_INT$_{MS}$ (Singlet)

Zero-point correction= 0.324272 (Hartree/Particle)
Thermal correction to Energy= 0.344664
Thermal correction to Enthalpy= 0.345609
Thermal correction to Gibbs Free Energy= 0.273433
Sum of electronic and zero-point Energies= -898.931333
Sum of electronic and thermal Energies= -898.910940
Sum of electronic and thermal Enthalpies= -898.909996

Sum of electronic and thermal Free Energies= -898.982171

C	-1.618600	-2.701800	-0.363300
C	-1.166100	-1.351600	-0.187000
C	-2.186500	-0.330000	0.088400
C	-3.562200	-0.700400	0.119300
C	-3.970100	-2.024500	-0.051100
C	-2.966600	-3.021500	-0.283300
H	-0.862700	-3.469200	-0.552200
H	-4.302500	0.085400	0.300500
H	-3.277500	-4.064300	-0.411600
C	-1.655500	0.962200	0.240900
C	-0.360400	1.318500	0.154200
C	0.598700	0.213700	-0.215000
C	0.241800	2.707300	0.497100
H	0.506400	2.727300	1.570800
H	1.181600	2.800500	-0.069800
N	0.146400	-1.058400	-0.352900
N	1.882700	0.575400	-0.407100
C	2.961500	-0.235200	-0.131800
C	4.218300	0.162200	-0.697300
C	2.937100	-1.406300	0.697500
C	5.373100	-0.597200	-0.489900
H	4.236700	1.066700	-1.311600
C	4.106500	-2.146700	0.910500
H	2.001100	-1.707300	1.168900
C	5.327100	-1.757800	0.316400
H	6.317900	-0.286100	-0.946700
H	4.072000	-3.034500	1.550500
H	6.234800	-2.343400	0.490600
C	-5.436600	-2.419900	0.004300
H	-5.626400	-3.151500	0.811400
H	-6.084100	-1.545900	0.182900
H	-5.761600	-2.893700	-0.940500
N	-0.619200	3.853900	0.218100
C	-1.802200	3.921000	1.067100
H	-2.375900	4.837500	0.851500
H	-2.463600	3.026800	0.891800
H	-1.506700	3.918600	2.129800
C	-0.908900	4.075700	-1.207600
H	-1.609900	3.318500	-1.623100
H	-1.359400	5.074300	-1.333300
H	0.029600	4.037400	-1.783000

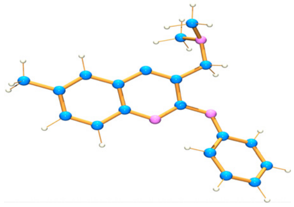

11_INT$_{MS}$ (Triplet)

Zero-point correction= 0.324984 (Hartree/Particle)
Thermal correction to Energy= 0.345300
Thermal correction to Enthalpy= 0.346245
Thermal correction to Gibbs Free Energy= 0.273119
Sum of electronic and zero-point Energies= -898.927767
Sum of electronic and thermal Energies= -898.907450
Sum of electronic and thermal Enthalpies= -898.906506
Sum of electronic and thermal Free Energies= -898.979631

C	-1.485900	-2.773600	-0.241600
C	-1.097600	-1.394900	-0.137600
C	-2.158000	-0.394200	0.008800
C	-3.515200	-0.820200	0.060100
C	-3.866000	-2.170500	-0.033800
C	-2.821900	-3.142100	-0.188400
H	-0.694100	-3.518300	-0.363400
H	-4.291800	-0.056900	0.172400
H	-3.092400	-4.200800	-0.267800
C	-1.707700	0.943500	0.081200
C	-0.406900	1.326800	0.022900
C	0.595900	0.234600	-0.091200
C	0.120800	2.789000	0.071600
H	0.769100	2.892300	0.959100
H	0.776800	2.938900	-0.802200
N	0.218700	-1.069900	-0.167100
N	1.892200	0.640700	-0.185200
C	3.002500	-0.163700	-0.059200
C	4.232200	0.412300	-0.528600
C	3.062700	-1.464500	0.550800
C	5.441000	-0.282600	-0.435900
H	4.184900	1.410500	-0.972200
C	4.285200	-2.136800	0.658800
H	2.147300	-1.913700	0.932400
C	5.477200	-1.563200	0.160600
H	6.361700	0.171400	-0.815500
H	4.316200	-3.121300	1.137000
H	6.424800	-2.103500	0.247400
C	-5.316100	-2.619700	0.020500
H	-5.490700	-3.310000	0.866600

H	-6.001100	-1.764100	0.135300
H	-5.602600	-3.162800	-0.899200
N	-0.871900	3.864700	0.102900
C	-1.679600	3.903400	1.324900
H	-2.275500	4.831800	1.343900
H	-2.377600	3.033600	1.392900
H	-1.019900	3.889600	2.208500
C	-1.683100	3.970200	-1.114100
H	-2.389300	3.112800	-1.225400
H	-2.270900	4.903500	-1.084100
H	-1.025200	3.996600	-1.998600

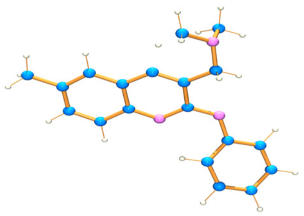

11_TS$_{\gamma HT}$

Zero-point correction= 0.321836 (Hartree/Particle)
Thermal correction to Energy= 0.341319
Thermal correction to Enthalpy= 0.342263
Thermal correction to Gibbs Free Energy= 0.273000
Sum of electronic and zero-point Energies= -898.931829
Sum of electronic and thermal Energies= -898.912347
Sum of electronic and thermal Enthalpies= -898.911403
Sum of electronic and thermal Free Energies= -898.980666

C	1.581600	-2.695500	-0.300200
C	1.133400	-1.339100	-0.153800
C	2.177900	-0.321100	0.072100
C	3.551400	-0.698400	0.063500
C	3.948700	-2.027800	-0.082300
C	2.930000	-3.021000	-0.251500
H	0.818300	-3.465700	-0.440500
H	4.301800	0.087300	0.202600
H	3.228000	-4.070500	-0.355400
C	1.648100	0.972400	0.202800
C	0.352100	1.322200	0.146100
C	-0.639800	0.231500	-0.201800
C	-0.182600	2.715000	0.538900
H	-1.099600	2.918600	-0.032800
H	-0.449900	2.722200	1.612500
N	-0.174900	-1.042600	-0.320100
N	-1.905200	0.621800	-0.357200
C	-3.009700	-0.181700	-0.122400
C	-4.257000	0.280500	-0.649800
C	-3.013000	-1.398400	0.632400
C	-5.434000	-0.455700	-0.473600
H	-4.257200	1.219800	-1.210300
C	-4.202700	-2.118100	0.814900
H	-2.080900	-1.759100	1.068800
C	-5.417800	-1.662000	0.261600
H	-6.372400	-0.088700	-0.902300
H	-4.184500	-3.044800	1.398400
H	-6.340600	-2.231100	0.410300
C	5.414300	-2.431300	-0.061800
H	5.708700	-2.931100	-1.003300
H	6.071600	-1.556500	0.073400
H	5.626800	-3.142000	0.758400
N	0.822900	3.756600	0.298000
C	2.037700	3.515600	1.006300
H	2.838800	4.220200	0.739100
H	1.880600	3.463500	2.096000
C	0.990300	4.160800	-1.112700
H	0.003000	4.386000	-1.543200
H	1.610400	5.070300	-1.153000
H	1.473500	3.369800	-1.721700
H	2.371500	2.392700	0.685600

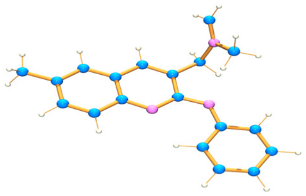

11_INT$_{\gamma HT}$

Zero-point correction= 0.326655 (Hartree/Particle)
Thermal correction to Energy= 0.346647
Thermal correction to Enthalpy= 0.347591
Thermal correction to Gibbs Free Energy= 0.276758
Sum of electronic and zero-point Energies= -898.975182
Sum of electronic and thermal Energies= -898.955191
Sum of electronic and thermal Enthalpies= -898.954247
Sum of electronic and thermal Free Energies= -899.025079

C	-2.271500	-2.293400	0.319900
C	-1.608800	-1.055200	0.014100
C	-2.447700	0.100900	-0.241600
C	-3.868600	-0.019700	-0.192700
C	-4.491000	-1.231900	0.103900
C	-3.656300	-2.371600	0.359400
H	-1.648300	-3.170600	0.515800

H	-4.476800	0.871500	-0.393300
H	-4.132900	-3.331100	0.592200
C	-1.767900	1.317200	-0.543900
C	-0.385200	1.361500	-0.595100
C	0.403400	0.146000	-0.305500
C	0.329700	2.644600	-0.969800
H	1.333500	2.405900	-1.356900
H	-0.236500	3.239500	-1.703600
N	-0.248100	-1.024200	-0.017100
N	1.746000	0.313800	-0.326000
C	2.710000	-0.679400	-0.165200
C	4.065800	-0.216700	-0.214000
C	2.510700	-2.086000	0.029600
C	5.152000	-1.088000	-0.069000
H	4.230700	0.852800	-0.381000
C	3.609400	-2.950000	0.171700
H	1.494200	-2.471100	0.069100
C	4.933800	-2.469100	0.127400
H	6.173300	-0.694000	-0.114200
H	3.426200	-4.020500	0.319100
H	5.779400	-3.155500	0.238000
C	-6.004300	-1.365800	0.162600
H	-6.344000	-1.700500	1.160700
H	-6.502800	-0.406800	-0.056700
H	-6.373700	-2.110800	-0.566800
N	0.514900	3.522200	0.236900
C	-0.199700	4.620300	0.413100
H	-0.087900	5.195400	1.332600
H	-0.918900	4.920200	-0.348900
H	-2.361200	2.216300	-0.759700
C	1.521700	3.091300	1.232300
H	2.521800	3.394600	0.878500
H	1.304700	3.559900	2.203100
H	1.517700	1.992200	1.293100

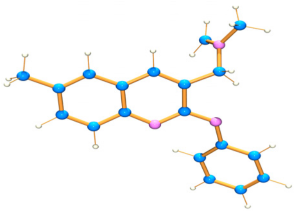

11_TS$_R$

Zero-point correction= 0.325830 (Hartree/Particle)
Thermal correction to Energy= 0.345256
Thermal correction to Enthalpy= 0.346200
Thermal correction to Gibbs Free Energy= 0.276889
Sum of electronic and zero-point Energies= -898.966915
Sum of electronic and thermal Energies= -898.947489
Sum of electronic and thermal Enthalpies= -898.946545
Sum of electronic and thermal Free Energies= -899.015856

C	1.851400	-2.649400	-0.084700
C	1.327400	-1.316600	-0.151700
C	2.278000	-0.226600	-0.163700
C	3.674900	-0.497500	-0.103600
C	4.168600	-1.803400	-0.036100
C	3.222000	-2.877400	-0.027300
H	1.137700	-3.478300	-0.082300
H	4.374600	0.348300	-0.116500
H	3.592700	-3.908000	0.023000
C	1.732500	1.097100	-0.231100
C	0.367200	1.286600	-0.280100
C	-0.535400	0.122900	-0.280500
C	-0.311600	2.647300	-0.377200
H	-0.420900	2.968800	-1.427000
H	-1.338200	2.534400	0.028400
N	-0.027700	-1.137900	-0.198000
N	-1.853500	0.417500	-0.433200
C	-2.897200	-0.453000	-0.164800
C	-4.169700	-0.103400	-0.724000
C	-2.842900	-1.621000	0.666200
C	-5.311100	-0.878100	-0.488000
H	-4.218900	0.791900	-1.351400
C	-3.996900	-2.381900	0.904700
H	-1.889700	-1.915500	1.103600
C	-5.236500	-2.026700	0.331200
H	-6.266400	-0.588400	-0.939000
H	-3.929300	-3.268200	1.545600
H	-6.129000	-2.631400	0.521100
C	5.660600	-2.093900	0.020100
H	5.990100	-2.699300	-0.845200
H	6.252100	-1.162900	0.020700
H	5.930100	-2.663000	0.929500
N	0.397900	3.746700	0.333700
C	0.735700	3.624400	1.613100
H	1.198900	4.464400	2.130200
H	0.571400	2.671300	2.113300
H	2.421700	1.951100	-0.224700
C	0.524100	5.037500	-0.371000
H	-0.476000	5.474900	-0.536100
H	1.130500	5.731200	0.228100
H	1.005500	4.873600	-1.348100

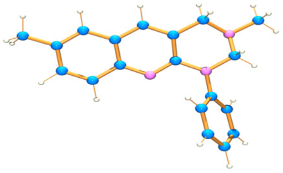

12

Zero-point correction= 0.330349 (Hartree/Particle)
Thermal correction to Energy= 0.349177
Thermal correction to Enthalpy= 0.350121
Thermal correction to Gibbs Free Energy= 0.282583
Sum of electronic and zero-point Energies= -899.028502
Sum of electronic and thermal Energies= -899.009674
Sum of electronic and thermal Enthalpies= -899.008729
Sum of electronic and thermal Free Energies= -899.076268

C	-2.526800	-1.888800	-0.237300
C	-1.801000	-0.665500	-0.095100
C	-2.551500	0.558700	0.043800
C	-3.977700	0.518500	0.037700
C	-4.674800	-0.682900	-0.099500
C	-3.917600	-1.890600	-0.238100
H	-1.956300	-2.815400	-0.347300
H	-4.529100	1.460900	0.143500
H	-4.453100	-2.840400	-0.349300
C	-1.792100	1.763200	0.173100
C	-0.409600	1.738600	0.178100
C	0.241400	0.450100	0.036700
C	0.423700	3.002400	0.321600
H	-0.085500	3.852300	-0.164600
H	0.539800	3.269000	1.404300
N	-0.428700	-0.698100	-0.100000
N	1.640000	0.411200	-0.009000
C	2.380000	-0.827900	0.009200
C	3.305100	-1.089100	-1.024300
C	2.232000	-1.756700	1.062300
C	4.082100	-2.262200	-1.000400
H	3.399000	-0.379000	-1.851500
C	3.001100	-2.933400	1.075100
H	1.506800	-1.560200	1.854900
C	3.931300	-3.189300	0.048100
H	4.793800	-2.456400	-1.809000
H	2.875900	-3.650300	1.892800
H	4.529900	-4.105200	0.063600
C	-6.195400	-0.729800	-0.106500
H	-6.584800	-1.358400	0.715800
H	-6.630300	0.277100	0.006600
H	-6.582400	-1.159200	-1.049300
N	1.743300	2.819900	-0.315500
C	2.406900	1.645000	0.258300
H	3.399300	1.536400	-0.202600
H	2.557600	1.763000	1.364900
H	-2.320300	2.720400	0.265100
C	2.583800	4.021000	-0.187400
H	2.801600	4.291700	0.873800
H	3.540800	3.860900	-0.711000
H	2.069800	4.873100	-0.661400

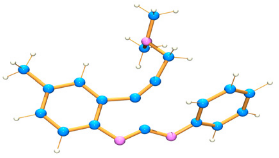

11_TS$_{DA}$

Zero-point correction= 0.323449 (Hartree/Particle)
Thermal correction to Energy= 0.343965
Thermal correction to Enthalpy= 0.344909
Thermal correction to Gibbs Free Energy= 0.272737
Sum of electronic and zero-point Energies= -898.914303
Sum of electronic and thermal Energies= -898.893787
Sum of electronic and thermal Enthalpies= -898.892843
Sum of electronic and thermal Free Energies= -898.965015

C	3.473300	-2.251800	-0.086400
C	2.120500	-1.865900	-0.075300
C	1.790000	-0.476500	-0.041400
C	2.785300	0.512100	-0.010100
C	4.148400	0.129900	-0.018200
C	4.464700	-1.251800	-0.051400
H	3.739400	-3.312000	-0.100600
H	2.504700	1.570100	0.004500
H	5.518300	-1.553300	-0.052100
C	0.327600	-0.333900	-0.086600
C	-0.563300	0.573100	-0.235300
C	-0.097800	-2.054300	0.051000
C	-1.155400	1.915400	-0.426200
H	-1.298100	2.093700	-1.508100
H	-2.167200	1.980200	0.039300
N	1.014900	-2.724700	-0.103200
N	-1.306200	-2.364800	0.425000
C	-2.412700	-1.622000	0.132500
C	-3.494000	-1.599700	1.074100

C	-2.537800	-0.839100	-1.067400
C	-4.631200	-0.834000	0.829900
H	-3.392400	-2.197700	1.983700
C	-3.718100	-0.090700	-1.308700
H	-1.814300	-0.979300	-1.872000
C	-4.749600	-0.069600	-0.365000
H	-5.445600	-0.823500	1.561000
H	-3.824300	0.455900	-2.251500
H	-5.658300	0.510300	-0.553000
C	5.251700	1.179800	-0.030400
H	4.864900	2.172700	0.254100
H	6.064700	0.920800	0.670900
H	5.709100	1.278600	-1.033200
N	-0.232700	2.954900	0.098300
C	-0.266400	3.020400	1.572100
H	0.487300	3.745000	1.920600
H	-1.264300	3.333300	1.962400
H	-0.020500	2.034600	1.995600
C	-0.507600	4.268500	-0.513200
H	-1.532300	4.649000	-0.285500
H	0.223200	5.001100	-0.133700
H	-0.392400	4.203300	-1.607600

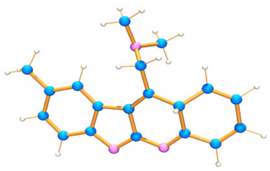

13_{DA}

Zero-point correction= 0.327633 (Hartree/Particle)

Thermal correction to Energy= 0.347015

Thermal correction to Enthalpy= 0.347959

Thermal correction to Gibbs Free Energy= 0.279446

Sum of electronic and zero-point Energies= -898.968225

Sum of electronic and thermal Energies= -898.948843

Sum of electronic and thermal Enthalpies= -898.947899

Sum of electronic and thermal Free Energies= -899.016412

C	3.222100	-2.420900	0.206500
C	1.892000	-1.978500	0.108000
C	1.599600	-0.582900	-0.061900
C	2.641900	0.359100	-0.108800
C	3.985700	-0.078200	-0.016900
C	4.252100	-1.463900	0.138200
H	3.440000	-3.484100	0.340000
H	2.416800	1.426300	-0.175000
H	5.293400	-1.796300	0.217500
C	0.134600	-0.520500	-0.148000
C	-0.785600	0.444900	-0.443100
C	-0.287300	-1.951300	0.039700
C	-0.403200	1.869200	-0.844500
H	0.350000	1.804900	-1.652400
H	-1.285500	2.391600	-1.284800
N	0.736100	-2.787200	0.167300
N	-1.598300	-2.366300	0.084800
C	-2.526900	-1.449200	-0.114700
C	-3.916400	-1.828200	0.071500
C	-2.245300	-0.005300	-0.585100
C	-4.906400	-0.882600	0.180600
H	-4.117300	-2.892500	0.222300
C	-3.339000	0.973400	-0.173300
H	-2.386700	-0.079800	-1.698900
C	-4.594500	0.539300	0.122900
H	-5.937900	-1.190300	0.378300
H	-3.119900	2.042600	-0.207200
H	-5.385200	1.263000	0.345600
C	5.133800	0.920200	-0.082400
H	4.765600	1.959000	-0.049300
H	5.837800	0.785400	0.758500
H	5.719200	0.804000	-1.013900
N	0.212000	2.675300	0.238300
C	-0.666800	2.859000	1.406200
H	-0.103700	3.375300	2.201200
H	-1.575800	3.466300	1.177900
H	-0.986900	1.879100	1.793200
C	0.676500	3.971200	-0.283600
H	-0.156500	4.611100	-0.665100
H	1.193400	4.523400	0.518700
H	1.390100	3.809600	-1.109300
